# A shoot endosymbiont colonizes pine host by unique and rhizobia-like mechanisms boosted by surface-fixed methanol

**DOI:** 10.1093/pcp/pcaf135

**Published:** 2025-10-24

**Authors:** Janne J Koskimäki, Johanna Pohjanen, Emmi-Leena Ihantola, Suvi Sutela, Anna Maria Pirttilä

**Affiliations:** Ecology and Genetics Research Unit, University of Oulu, PO Box 3000, Pentti Kaiteran katu 1, FI-90014 Oulu, Finland; Ecology and Genetics Research Unit, University of Oulu, PO Box 3000, Pentti Kaiteran katu 1, FI-90014 Oulu, Finland; Ecology and Genetics Research Unit, University of Oulu, PO Box 3000, Pentti Kaiteran katu 1, FI-90014 Oulu, Finland; Ecology and Genetics Research Unit, University of Oulu, PO Box 3000, Pentti Kaiteran katu 1, FI-90014 Oulu, Finland; Forest Health and Biodiversity, Natural Resources Institute Finland (Luke), Latokartanonkaari 9, FI-00790 Helsinki, Finland; Ecology and Genetics Research Unit, University of Oulu, PO Box 3000, Pentti Kaiteran katu 1, FI-90014 Oulu, Finland

**Keywords:** *Methylorubrum*, endophyte, endosymbiont, plant–microbe interaction, methanol utilization, MxaF

## Abstract

*Methylorubrum extorquens* DSM13060 (Rhizobiales) has a specific capacity to live inside cells of bud meristems in pine trees. The bud niche is almost completely unstudied, although likely widespread in plants. It is unknown how the endosymbiotic methylotroph enters such crucial tissues of the plant. We hypothesized the bud colonization to occur mainly through the shoot epidermis enabled by host-produced methanol. We combined several microscopic methods to illustrate spatio-temporal colonization dynamics and methanol utilization by *M. extorquens* DSM13060 during the interaction. Our results showed that the endosymbiont mainly enters pine seedlings through cylindrical sheath, which is a layer of living cells surrounding primary root and transition zone. The cylindrical sheath played a central role in accumulation and proliferation of bacteria before entering deeper tissues. The endosymbiont also penetrated host through epidermis and stomatal apertures in stem and formed infection pocket-like structures upon entry. *M. extorquens* DSM13060 activated the *mxaF*-promoter on plant surfaces for methanol assimilation prior to shifting to the endosymbiotic lifestyle. Our results suggest that the surface-bound methanol was used for production of antioxidants that enable tissue penetration, documented earlier. Gradual cell-to-cell passage or formation of intracellular infection threads enabled the invasion past endodermis into the xylem. The xylem was observed to function as the main route to the apical meristem, where bacteria were present after 90 days of inoculation. Our study widens the previously known niches and reveals unique and rhizobia-like colonization mechanisms by the endosymbiont in the above and belowground parts of pine.

## Introduction

Scots pine (*Pinus sylvestris* L.) has the widest distribution range of all pines, extending from Western Europe to Eastern Siberia and from Southern Spain beyond the Arctic Circle in Fennoscandia (Matías and Jump 2012). Behind the successful colonization of a harsh habitat by subarctic plant species lies a vast interplay with symbiotic organisms: mycorrhizal fungi, rhizospheric bacteria, and microbes that live asymptomatically inside the plant tissue—endophytes (Harrison 1999, Hardoim et al. 2015). Symbiosis between bacterial endophytes and plants is both widespread and necessary for plant health and survival (Ryan et al. 2008, Taghavi et al. 2009). Endophytes are important for plant defense, stress tolerance, and promotion of plant growth and development (Hardoim et al. 2015). However, a majority of studies describe endophytic interactions in model plant species or agricultural crops (Ryan et al. 2008, Izumi 2011, Hardoim et al. 2015). Bacterial endophytes may provide significant host benefits and increased phenotypic plasticity in the long-living forest trees that face variable environmental conditions (Carrell and Frank 2014, Hardoim et al. 2015).

*Methylorubrum extorquens* DSM13060 (formerly *M. extorquens*) is not only an endophyte but also an endosymbiont, isolated from the meristematic cells of shoot tips of Scots pine (Pirttilä et al. 2000). Endosymbionts are microbes that reside inside a host cell (McCutcheon et al. 2019), whereas endophytes usually occupy the space between cells, most typically in plant roots (Ryan et al. 2008, Izumi 2011, Hardoim et al. 2015). The facultative, methylotrophic endosymbiont, *M. extorquens* DSM13060, represents the dominant species in pine meristems throughout the year, detected prior to bud elongation and development but not during active growth (Pirttilä et al. 2005). In general, methylotrophic bacteria, which utilize one-carbon compounds for energy (Chistoserdova and Lidstrom 2013), are abundant in the phyllosphere, where they assimilate plant-produced methanol (Fall and Benson 1996, Delmotte et al. 2009). *M. extorquens* DSM13060 fixes the methanol into polyhydroxybutyrate (PHB) (Koskimäki et al. 2016), which is known as a bacterial carbon and energy reserve compound (Bourque et al. 1995, Ratcliff et al. 2008). However, upon host infection, *M. extorquens* DSM13060 depolymerizes the product into short oligomers that possess strong antioxidant activity (Koskimäki et al. 2016). Similar to *M. extorquens* DSM13060, rhizobia produce and utilize PHB during infection of legume roots (Hirsch et al. 1983).

*M. extorquens* DSM13060 increases lateral root formation, root length, and aboveground biomass in the host without producing any well-known plant hormones (Pirttilä et al. 2004) to the same extent as ectomycorrhizal fungi (Pohjanen et al. 2014). The endosymbiont aggregates near the nuclei of living cells and modulates host functions potentially by eukaryote-like effectors (Koskimäki et al. 2015). The interaction results in induced development and viability of the host through mechanisms previously unknown for endophytic bacteria; intrinsic manipulation of hormonal pathways, down-regulation of senescence and cell death-associated genes, and induction of ononitol biosynthesis (Koskimäki et al. 2022). The interaction is highly mutualistic and novel compared to plant-endophyte interactions reported earlier, mainly from crop plant roots (Izumi 2011). However, the details on how *M. extorquens* DSM13060 enters the buds have remained unknown. We hypothesized the bud colonization to occur mainly through shoot epidermis, enabled by fixing of host-produced methanol. We used a dual labeling strategy to illustrate the spatio-temporal dynamics of pine colonization and methanol utilization by *M. extorquens* DSM13060. Our study reveals the first steps (0–730 days) in the symbiotic relationship between the endosymbiont and the economically important conifer, Scots pine.

## Results

The pattern of endophytic colonization was consistent between the inoculated samples (*n* = 5) throughout the experiment. The host plants were visually healthy after inoculation, as before (Pohjanen et al. 2014), and the phenotypes of the reporter strains *M. extorquens* 13061 and *M. extorquens* 13061-*mxaF* remained unchanged compared to the wild-type strain. The fluorescent reporters, GFP and mCherry, were selected to minimize bleed-through effects and to avoid overlapping of the fluorescent wavelengths. The combination of these reporters, two consecutive chromosomal copies of GFP and mCherry in a stable, low-copy plasmid, created fluorescent emissions that were clearly distinguishable from the plant tissues. Endogenous autofluorescence of plant tissue was detected in the third laser channel (488 nm/LP 650 nm) and used to visualize the background.

### Colonization of roots

**Figure 1:**
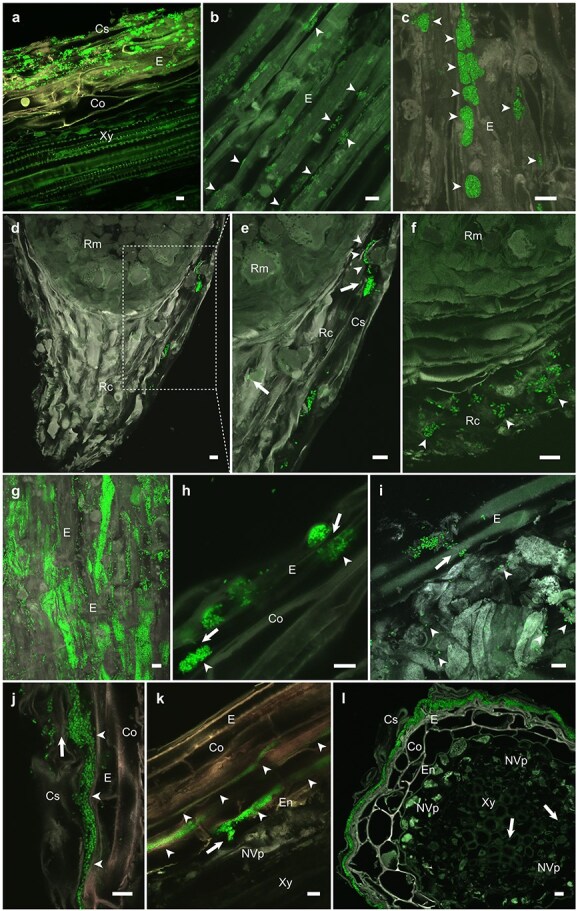
Colonization of *P. sylvestris* L. roots by *M. extorquens* DSM13060. Images of longitudinal and cross sections of root tissues at 7–110 days post-inoculation (dpi). Bacterial cells carrying a fluorescent GFP are visible in bright green. (a) Bacterial colonies and individual cells in the cylindrical sheath and epidermis at 7 dpi. (b) Bacteria invaded cells from gaps between the cells of root epidermis (arrowheads) at 15–30 dpi and (c) eventually enlarged into infection pocket-like structures at 30–50 dpi. (d) Infection pocket-like structures were observed in the cylindrical sheath covering root tip at 40–60 dpi (e) from where bacterial colonization progressed vertically (arrowheads). The square in (d) represents magnified area. (f) During 30–50 dpi bacteria were abundant also in the root cap. (g) Infection pocket-like structures proliferated into larger biofilm-like colonies, partially on the surface, and within the cells of cylindrical sheath and root epidermis at 50–80 dpi. (h) From the infection pocket-like structures, bacteria penetrated cell walls into neighboring cells. The arrows indicate penetration sites and the arrowheads established endophytic colonies initiating the vertical invasion. (i) A funnel-like penetration site (arrow), puncturing the epidermis by an abundance of bacteria (arrowheads) into the adjacent cortical cells. (j) Biofilm-like bacterial growth (arrowheads) between the layers of cylindrical sheath and epidermis, derived from the root surface (arrow). (k) Bacteria formed infection-thread-like structures (arrowheads) to spread horizontally through the inner cortex and to penetrate (arrow) non-vascular parenchyma at ≥90 dpi. (l) Biofilm-like bacterial growth eventually covering the whole root at 80–100 dpi. The arrows indicate bacterial cells in the xylem vessels. Microscopic sections: longitudinal (a–k); cross (l). Co, cortex; Cs, cylindrical sheath; E, epidermis; En, endodermis; NVp, non-vascular parenchyma; Rc, root cap; Rm, root meristem; Xy, xylem. Dashed square = magnified area. Scale bars; 10 μm.

**Figure 2:**
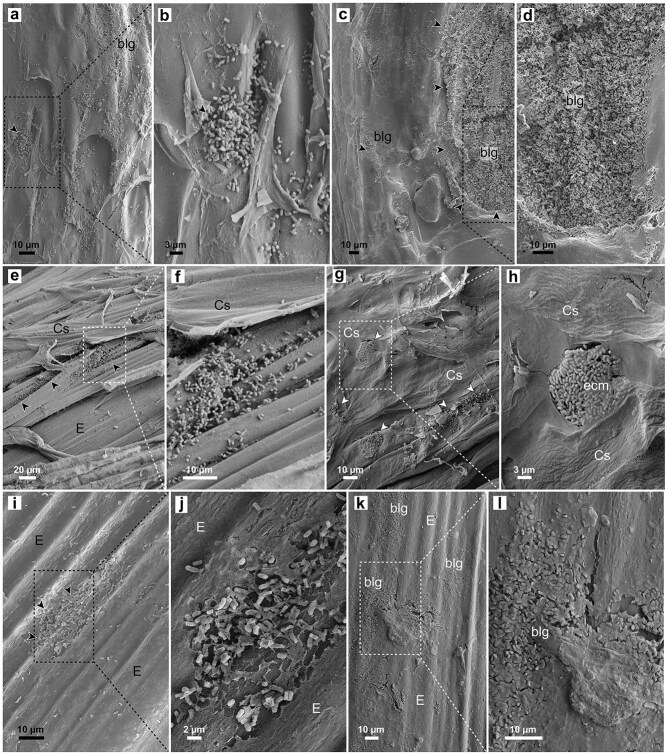
Colonization of *Pinus sylvestris* L. root and shoot surfaces by *Methylobacterium extorquens* DSM13060. FE-SEM images of pine roots at 80–100 days post-inoculation (dpi) and 730 dpi. (a) Bacterial biofilm-like growth on the root surface, near the root tip, with (b) round-shaped aggregate over putative penetration site at 80–100 dpi. (c) Smooth appearance of the root surface reveals thick bacterial growth embedded in the mucilagous sheath at 730 dpi. (d) Magnification shows the abundance of bacteria in the root mucilage. (e) Bacterial growth on the surface of lower stem with remnants of cylindrical sheath. (f) Bacteria entering the cracks between the cylindrical sheath and epidermis. (g) Bacterial colonies on the stem and (h) a round-shaped microcolony, partially covered by extracellular matrix (ecm), at a putative penetration site. (i) Sparse bacterial growth on smooth surface of the shoot, lacking cylindrical sheath. (j) Bacterial colony penetrating through shoot epidermis at a putative penetration site. (k and l) Large biofilm-like (blg) structures on the shoot surface at 730 dpi. blg, biofilm-like growth; Cs, cylindrical sheath; E, epidermis; ecm, extracellular matrix. Dashed square = magnified area.

Bacterial colonization of pine seedlings started from the roots. At 7 dpi, individual bacterial cells and small colonies were observed on the root surface. *M. extorquens* DSM13060 formed structures that resemble the infection pockets of stem-colonizing rhizobia (Goormachtig et al. 2004) 7–14 dpi in the cylindrical sheath, which is a living tissue that covers the epidermis of the primary root (Fig. 1a, Tillman-Sutela et al. 2008). The colonization had extended into root epidermis (Fig. 1b and c; Supplementary Video S1) and infection pocket-like structures were visible in the root cap at 40–60 dpi (Fig. 1d–f; Supplementary Video S2). Bacteria heavily colonized on the root surface and in the cells of cylindrical sheath and root epidermis at 50–80 dpi (Figs 1g and 2a,b). The bacterial cells were observed penetrating directly through plant cell walls (Fig. 1h and i) and expanding vertically (Fig. 1j). Vertical colonization was also evident in the inner cortex (Fig. 1k; Supplementary Fig. S1a–c; Supplementary Video S3), until the bacteria reached the vascular tissues. Although the colonization was mainly progressing toward the stele, at 80–100 dpi the bacteria formed a thick biofilm-like structure between cylindrical sheath and root epidermis, covering the whole primary root (Fig. 1l). In the final sampling at 730 dpi, the majority of bacteria were localized in the cylindrical sheath (Fig. 2c and d) and persistently in the parenchymatous cells of xylem (Supplementary Fig. S1d–f).

### Transition zone

The cylindrical sheath, which covers the roots and extends to the lower part of the stem in the transition zone (Supplementary Fig. S2a), had a key role in the vertical colonization by *M. extorquens* DSM13060. The morphology of the cylindrical sheath, having elongated unparallel cells in diverse orientations (Supplementary Fig. S2b), provided numerous cellular gaps and encasements for bacterial colonization (Fig. 2e and f; Supplementary Fig. S2c and d). Infection pocket-like structures were observed specifically in the belowground parts of the cylindrical sheath, being distinct in the cross-sections of the transition zone (Supplementary Fig. S2e). As soon as the cylindrical sheath was completely filled with bacteria, the colonization occurred toward the cortex (Supplementary Fig. S2f). At 60 dpi, *M. extorquens* DSM13060 was most abundant at the transition zone compared to roots, stem, or needles (Supplementary Table S3).

### Colonization of stem

**Figure 3:**
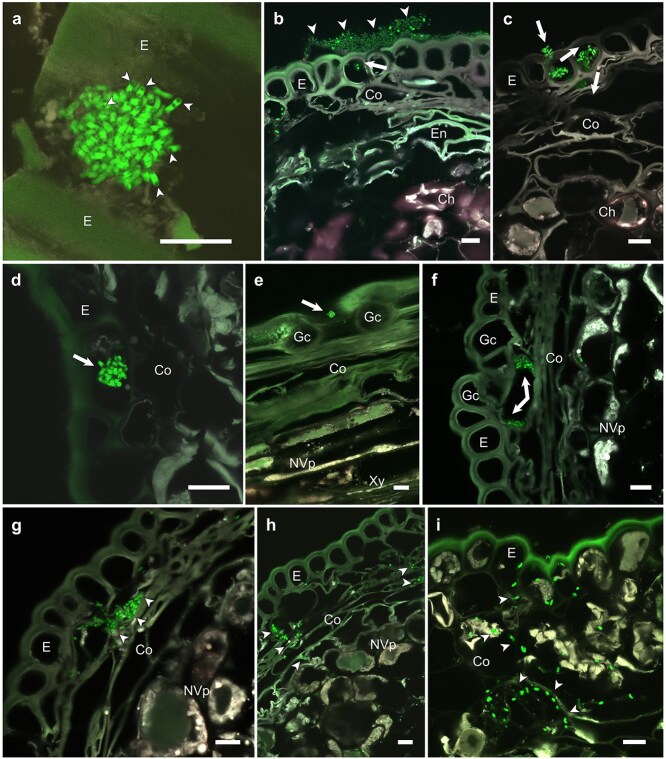
Colonization of the *P. sylvestris* L. shoots by *M. extorquens* DSM13060. CLSM images of longitudinal and cross sections of shoot tissues at 40–120 days post-inoculation (dpi). Bacterial cells carrying a fluorescent GFP are visible in bright green. (a) A round-shaped bacterial aggregate on the shoot surface at a putative entry point. Dark inclusions in the bacterial cells (arrowhead) are endogenous PHB granules. (b) Thick, raft-like bacterial colonies (arrowhead) consisting mainly of dead cells (no GFP fluorescence) and few live cells on the entry points of epidermis typically found on the shoots lacking cylindrical sheath at 80–100 dpi. An arrow depicts bacteria inside an epidermal cell. (c) After entry to epidermal cells (arrows) (d) bacteria formed tight aggregates (arrow), infection pocket-like structures, before invasion of the adjacent cells. (e) A bacterial aggregate (arrow) in the stomatal aperture at 80–100 dpi and (f) larger colonies in the stomatal airspaces (arrows) at 100–120 dpi. (g) Bacteria invaded the neighboring cells primarily vertically (arrowheads) and (h, i) eventually horizontally (arrowheads) toward the vascular tissues. Microscopic sections: longitudinal (a, e); cross (b–d, f–i). Ch, chlorenchyma; Co, cortex; Cs, cylindrical sheath; E, epidermis; En, endodermis; Gc, guard cell; NVp, non-vascular parenchyma; Xy, xylem. Scale bars: 10 μm.

The frequency of endophytic colonization was much lower on the stem than in the lower parts; transition zone and roots encased by the cylindrical sheath (Supplementary Table S3) after 80 dpi. Round-shaped microcolonies of *M. extorquens* DSM13060 were first observed on the lower stem surface ≥40 dpi (Figs 2g and h and 3a) and ≥ 80 dpi on the upper stem (Fig. 2i and j). At 80–100 dpi, these colonies formed a dense bacterial growth, eventually consisting of mainly dead cells at the sites of penetration (Fig. 3b). The morphology of the raft-like, thick microcolonies (arrowheads in Fig. 3b) on stem clearly differed from colonies previously observed on the roots. Once inside (Fig. 3c), bacterial cells propagated in the infection pockets below the epidermis (Fig. 3d) before spreading to the neighboring cells. Bacterial aggregates were detected in stomatal apertures (Fig. 3e), and later (100–120 dpi), large colonies were observed in the stomatal airspaces (Fig. 3f) and in the cortex beneath (Fig. 3g and h). Similar to roots, colonization of the stem occurred first mainly in vertical direction into the adjacent cells (Fig. 3g) and then horizontally toward the vascular tissues (Fig. 3h and i). At 730 dpi, bacteria were detected on the stem surfaces as an extensive biofilm-like growth (Fig. 2k and l), similar to roots.

### Route to the apex

**Figure 4:**
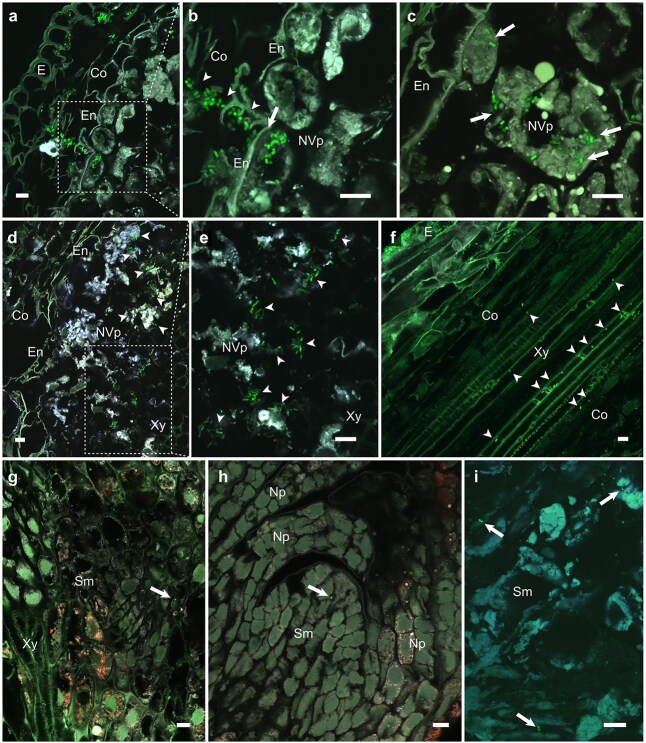
Systemic colonization of the *P. sylvestris* L. by *M. extorquens* DSM13060. CLSM images of longitudinal and cross sections of shoot tissues in the transition zone at 40–120 days post-inoculation (dpi). Bacterial cells carrying a fluorescent GFP are visible in bright green. (a) Bacteria formed infection thread-like structures to invade through the cortex and endodermis. The square represents magnified area. (b) Infection thread-like structure (arrowheads) of bacteria penetrating endodermis (arrow). (c) Bacteria invading host cell interior (arrows). (d, e) Aggregates of dividing bacteria (arrowheads) in the parenchymatous host cells surrounding the vascular tissues at 90–120 dpi. The square depicts the magnification area represented in (e). Note the enlarged bacterial cell morphology. (f) In the roots, individual bacterial cells (arrowheads) were evident inside the xylem vessels from 40 dpi onwards. (g) A bacterial cell (arrow) near the xylem vessels inside a meristematic cell of the shoot tip at 80–100 dpi. (h) A bacterial cell (arrow) in the shoot apical meristem (Sm) at 80–100 dpi. (I) In the apex, bacteria (arrows) resided inside the cytoplasm of meristematic cells high with endogenous fluorescing metabolites (at ≥100 dpi). Microscopic sections: longitudinal (f); cross (a–e, g–i). Co, cortex; E, epidermis; En, endodermis; Np, needle primordia; NVp, non-vascular parenchyma; Sm, shoot apical meristem; Xy, xylem. Dashed square = magnified area. Scale bars: 10 μm.

Besides the gradual cell-to-cell infection of the host cortex, we identified an alternative mode of invasion. From the infection pocket-like structures, *M. extorquens* DSM13060 formed infection thread-like structures passing through cortex and endodermis (Fig. 4a and b) into the vascular tissues. Such structures were also observed by *in situ* hybridization in buds of mature trees, advancing from scale primordia toward the apical meristem (Supplementary Fig. S3). In the seedlings, bacteria were often observed inside the cells of root vascular parenchyma (Fig. 4c) and in the photosynthetic chlorenchyma of the stem. Penetration of these cells occurred consistently by bacterial congregation as round-shaped clusters on the plant cell wall, followed by permeation of host cell by a successive thin bacterial thread into the host cytosol. The observed penetration sites were roughly of the diameter of a single bacterial cell (1–2 μm) and occurred without observed damage or cell death induction in the colonized tissue (See “Viability of colonized tissue” and Supplementary Fig. S4). From 90 dpi onwards, dividing bacterial cells were apparent in the vascular parenchyma (Fig. 4d and e). After the first weeks (>40 dpi) of colonization, individual bacterial cells were seen inside the root xylem vessels (Fig. 4f; Supplementary Video S4), which function as the most probable path to the shoot tips. Accordingly, despite high autofluorescence of apical tissues, we could repeatedly detect bacterial cells in the buds at ≥90 dpi (Fig. 4g and h) residing near the xylem vessels. In the buds, most of the bacteria colonized the cytoplasm of meristematic cells (Fig. 4i). Throughout the experiment, bacteria were not detected on the epidermis of buds (apex or needle primordia of seedlings).

### Viability of colonized tissue

We evaluated potential cell death induction by *M. extorquens* DSM13060 in the pfine tissues by dual acridine orange—ethidium bromide (AO-EB) staining. In AO-EB staining, healthy cells have a round green nucleus, early programmed-cell-death (PCD) cells have a green-yellow nucleus with condensed or fragmented chromatin, late-PCD cells display condensed and fragmented orange chromatin, and necrotic cells have a structurally normal, orange-red nucleus (Supplementary Fig. S5a–d). The endosymbiont colonized healthy cells when residing on root surfaces, occurring intracellularly near host nuclei (Supplementary Fig. S5e), during infection thread (Supplementary Fig. S5f), and infection pocket formation in the root cortex (Supplementary Fig. S5g). The bacteria also colonized inside living cells of the cylindrical sheath and non-vascular parenchyma (Supplementary Fig. S5h). At the transition zone, large bacterial aggregates colonized entire cells of non-vascular parenchyma next to living endodermal cells (Supplementary Fig. S5i), and in the cortex, bacteria colonized the cytoplasm of living cells (Supplementary Fig. S5j).

### Bacterial *mxaF* activity

**Figure 5:**
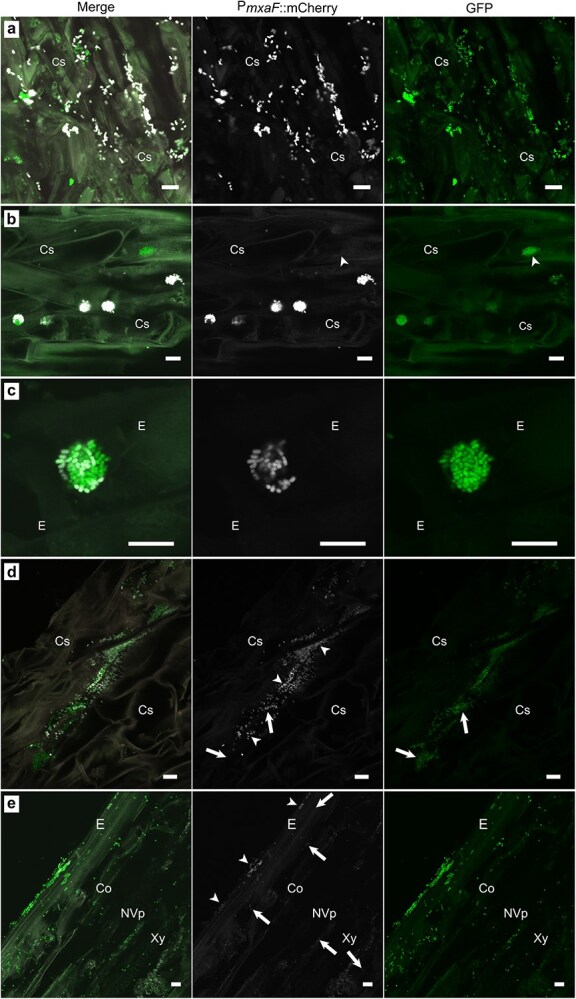
A reporter gene analysis of *M. extorquens* DSM13060 methanol assimilation during root colonization of *P. sylvestris* L. An mCherry reporter controlled by the methanol-inducible *mxaF* promoter (P*mxaF*::mCherry) was used as a biological sensor for bacterial methanol utilization. Each panel includes a merged image of pine tissue infected by bacteria carrying both tags, an image showing only mCherry (white), and only GFP tag (bright green). (a) Bacteria colonizing the cylindrical sheath at 7 dpi. (b) Round-shaped bacterial colonies in the cylindrical sheath of the root, displaying high-level methanol assimilation ≥14 dpi. The arrowhead depicts a colony without mCherry activity. (c) Unequal methanol assimilation within a bacterial colony on the root epidermis (at 14 dpi). (d) Bacteria in the infection pocket-like structures of root cylindrical sheath display higher methanol assimilation closer to surface (arrowheads) than deeper in the cell (arrows) (≥ 50 dpi). (e) Bacteria on the epidermis (arrowheads) exhibit higher methanol assimilation than bacterial cells colonizing the cortex or the stele (arrows) (≥50 dpi). Microscopic sections: longitudinal (a–c, e); cross (d). Co, cortex; Cs, cylindrical sheath; E, epidermis; NVp, non-vascular parenchyma; Xy, xylem. Scale bars: 10 μm.

We used the promoter of methanol dehydrogenase gene (*mxaF*) controlling a fluorescent reporter (mCherry) as a biological sensor for methanol utilization by the endosymbiont during colonization. Upon formation of the first bacterial microcolonies on the cylindrical sheath, high level of mCherry fluorescence was observed at 7–14 dpi (Fig. 5a–c). The bacterial methanol consumption was not constitutive, assessed by the varying *mxaF* promoter activity between individual bacterial cells and neighboring colonies (Fig. 5b and c). Aggregation into microcolonies often correlated with *mxaF* activity (Fig. 5b and c; Supplementary Fig. S6a and b), and the active bacterial aggregates were typically observed close to plant surfaces. The bacterial colonies residing deeper in the tissue had low or undetectable *mxaF* expression (Fig. 5d and e). For example, a bacterial infection pocket-like structure of an epidermal cell showed more *mxaF* promoter activity closer to surface than deeper within the cell (Fig. 5d). Similarly, at a later stage (≥60 dpi), biofilm-like structures between the cylindrical sheath and epidermis exhibited higher *mxaF* activity than bacterial cells located in the cortex or stele (Fig. 5e).

**Figure 6:**
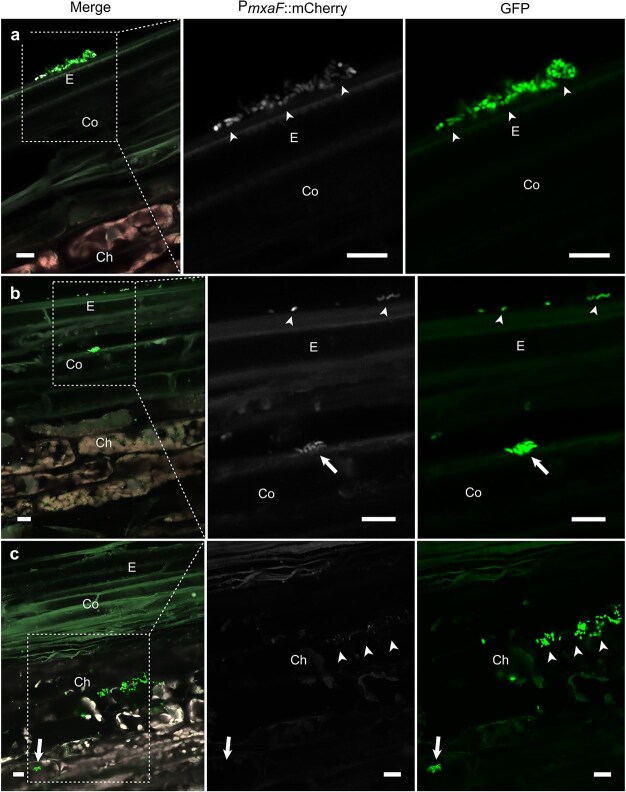
A reporter gene analysis of *M. extorquens* DSM13060 methanol assimilation during the shoot colonization of *P. sylvestris* L. An mCherry reporter controlled by the methanol-inducible *mxaF* promoter (P*mxaF*::mCherry) was used as a biological sensor for bacterial methanol utilization. CLSM images of longitudinal sections of shoot tissues at 80–120 days post-inoculation (dpi). Each panel includes a merged image of pine tissue infected by bacteria showing both tags, an image showing only mCherry (white), and only GFP tag (bright green). (a) Bacterial colonies on the shoot epidermis (arrowheads) displayed intermediate-level methanol assimilation, (b) and the expression was also within detectable range in the outer cortex (arrow). (c) In the inner cortex, bacterial methanol utilization was low (arrowheads) or non-existing during intracellular colonization of the chlorenchymal cell (arrow) beside vascular tissue. Ch, chlorenchymal cells; Co, cortex; E, epidermis; Xy, xylem. Dashed square = magnified area. Scale bars: 10 μm.

**Figure 7:**
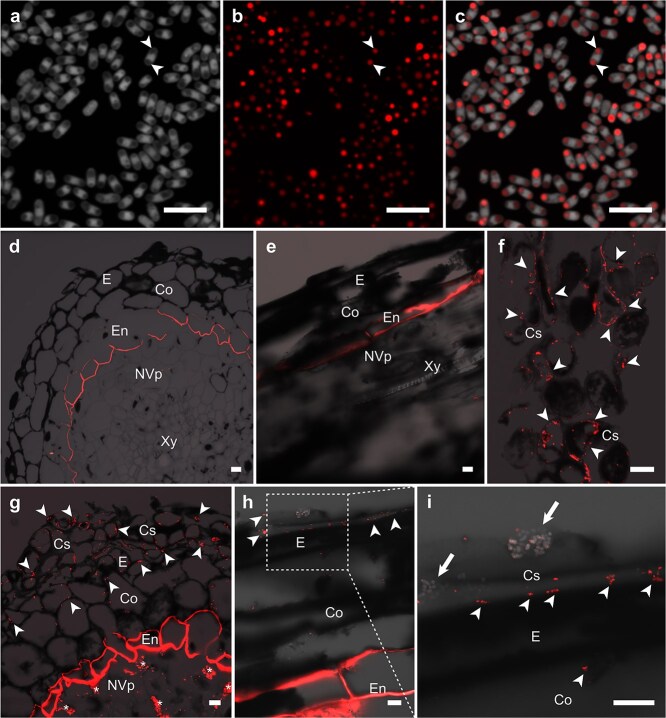
*M. extorquens* DSM13060 accumulates intracellular PHB storages during the early steps of pine colonization. CLSM of *M. extorquens* cells and scots pine root at 60 dpi. Bacterial PHB granules and pine endodermis are stained with Nile blue A (red). Endogenous autofluorescence of the plant cell walls is shown as a black silhouette, outlining root morphology. (a) Cultured bacterial cells (light gray) have dark inclusions without fluorescence (arrowheads). (b) Intracellular PHB granules stained with Nile blue a (arrowheads), and (c) a merged image of both laser channels, demonstrating that dark inclusions in (a) are PHB granules (arrowheads). (d) A cross-section of mock-inoculated control root without bacteria. (e) A longitudinal section of mock-inoculated control root without bacteria. (f) A section of cylindrical sheath colonized by bacteria carrying PHB granules. (g) Pine root inoculated with *M. extorquens* DSM13060 with numerous fluorescing PHB granules in bacteria colonizing the cylindrical sheath and epidermal cells. Asterisks depict putative starch granules in parenchymal cells of xylem. (h) A section of pine root where PHB granules of *M. extorquens* DSM13060 (arrowheads) are visible in the cells of epidermis. The square represents magnified area. (i) The arrows indicate clusters of bacterial cells (light gray) carrying the PHB granules, colonizing cylindrical sheath. Gamma levels of the micrographs were adjusted slightly to optimize image contrast and brightness for the dimmer PHB granules. Adjustment was applied equally across the images (d–i). Microscopic sections: longitudinal (e, h, i); cross (d, f, g). Co, cortex; Cs, cylindrical sheath; E, epidermis; En, endodermis; NVp, non-vascular parenchyma; Xy, xylem. Dashed square = magnified area. Scale bars, 10 μm.

In the shoots, strong activation of *mxaF* promoter was observed in *M. extorquens* DSM13060 residing on the epidermis (Fig. 6a and b; Supplementary Fig. S6c), and the expression was also detectable in the cortex (Fig. 6b). However, in the vascular parenchyma, *mxaF* activity was scarce or undetectable (Fig. 6c; Supplementary Fig. S6d). We observed dark spherical inclusion bodies within the GFP-tagged bacterial cells residing on the plant shoot surface (Fig. 3a) that were verified by Nile Blue A as PHB granules (Fig. 7a–c) that act as a reserve for carbon, energy, and antioxidative power (Bourque et al. 1995, Ratcliff et al. 2008, Koskimäki et al. 2016). When compared with the uninoculated control (Fig. 7d and e), the majority of the bacterial PHB granules were detected in the cylindrical sheath, epidermis, and outer cortex (Fig. 7f–i), thus correlating with the bacterial *mxaF* activity.

## Discussion

In general, knowledge on the lifestyles of bacterial endophytes in forest trees is limited (Izumi 2011, Pirttilä and Frank 2018). While most studies report on the endophytic diversity, host colonization is less studied, especially in woody species (Germaine et al. 2004, Rincón et al. 2005, Weyens et al. 2012). This is partly due to technical challenges such as high background autofluorescence of woody plant tissue that is rich with phenolic compounds (Timonen 1995, Anand and Chanway 2013). We tackled this issue by using a reproducible in vitro-inoculation system in combination with a mild fixation process and cryosectioning. The combination of fluorescent proteins with high intensity and minimal overlap of excitation and emission spectra enabled fluorophore detection from the host background noise for obtaining a comprehensive view of *M. extorquens* DSM13060 colonization.

### Endosymbiont colonization is unique but resembles rhizobial colonization

**Figure 8:**
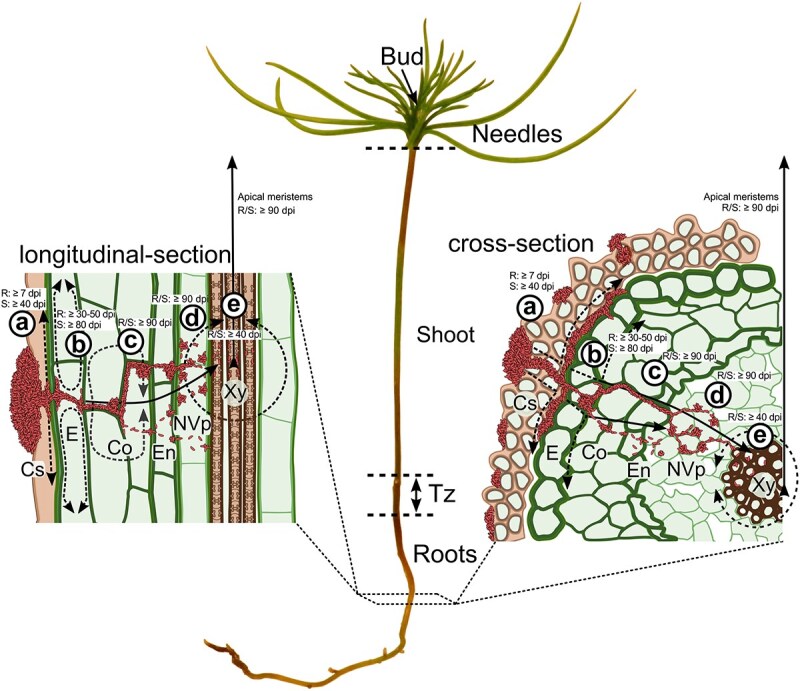
A simplified model of pine colonization by *M. extorquens* DSM13060, with a representation of the seedling anatomy and illustrations of the morphologies of the tissues. (a) At ≥7 dpi, the bacteria initiate pine colonization from the surface by forming microcolonies on cylindrical sheath or epidermis. At this stage, plant-derived methanol is utilized as the carbon source and stored as endogenous PHB granules to fuel plant invasion. (b) From 30–50 dpi onwards, the bacteria form infection pocket-like structures, penetrate epidermal cell walls and establish biofilm-like structures between the cylindrical sheath and root epidermis (dashed arrows). Once inside the plant tissues, the bacteria form multicellular aggregates to invade neighboring cells of epidermis and outer cortex (dashed arrows), eventually colonizing the surrounding host tissues. (c) At ≥90 dpi, gradual cell-to-cell-passage (single bacterial cells), or formation of intracellular infection thread-like structures (multicellular bacterial threads) through the cortex (arrow) enables the invasion of vascular tissues past endodermis. (d) At ≥90 dpi, the bacteria form intracellular aggregates and proliferate in the parenchymal cells around the xylem vessels (dashed circle). (e) From 90 dpi onwards, after accessing the xylem vessels (≥40 dpi), the systemic bacterial colonization occurs through the transpiration stream, reaching the apex. Cs, cylindrical sheath; Co, cortex; E, epidermis; En, endodermis; NVp, non-vascular parenchyma; Xy, xylem vessels.

Our study shows that *M. extorquens* DSM13060 colonization extends the previously known niche of plant buds to all seedling tissues. The roots were the main access point for the endosymbiont in pine seedlings (Fig. 8), similar to many endophytes and rhizobia. These microbes enter plants through cracks formed during root growth, such as lateral-root emergence sites (James et al. 1997, Dong et al. 2003, Capoen et al. 2010), besides the rhizobial root-hair-based colonization (Capoen et al. 2010). However, *M. extorquens* DSM13060 was not abundant at the sites of lateral root emergence, scarcely in root hairs or at the root hair zone, but it was detected in the root cap and at the root elongation and differentiation zones, inside and between the cortical cells at 7–60 dpi. The cylindrical sheath covering the hypocotyl was rich with bacteria especially at the transition zone, whereas the upper stem that lacks the sheath was colonized slowly. Our results emphasize the role of cylindrical sheath in the bacterial colonization, which is a unique feature among plant–microbe interactions.

Overall, the endosymbiotic colonization resembled that of stem-colonizing rhizobia in water-tolerant legumes (Capoen et al. 2010, Bonaldi et al. 2011). *M. extorquens-*colonies were observed on the stem in stomatal apertures and substomatal chambers, from where the colonization of vascular tissue occurred via intracellular infection thread-like-structures. Whereas stem-nodulating rhizobia form infection threads from epidermal cracks for advancing in the stem (Capoen et al. 2010), endophytes have previously been detected only in the leaf substomatal chambers (Compant et al. 2005). Our findings thus highlight the importance of stomata in stem for the endosymbiont colonization.

Stem-colonizing rhizobia trigger cell death within cortex to form intercellular infection pockets (D’Haeze et al. 1998). The endosymbiont formed infection pocket-like structures inside living cells of cortex and cylindrical sheath for further colonization of xylem through the thread-like structures, or by gradual cell-to-cell invasion (Fig. 8). There was no cell death observed with the endosymbiotic colonization process. Whereas colonization by rhizobia initiates cell divisions for nodule meristem development (Capoen et al. 2010), the endosymbiont colonizes bud meristems for manipulating plant development (Pirttilä et al. 2000, Pohjanen et al. 2014, Koskimäki et al. 2022). Genome of *M. extorquens* DSM13060 hosts *Nod-like* genes that encode for Nod factors, which are necessary for rhizobial colonization (Koskimäki et al. 2015). This supports the similarities found in colonization, although Nod factors have not been identified in *M. extorquens* DSM13060 (Pirttilä et al. 2004).

The majority of endophytes can spread only within the root cortex (James et al. 1994), and for accessing the shoots, a method of transportation is required. Ever since the discovery of bacteria in xylem vessels (Bell et al. 1995), the xylem and its transpiration stream have been considered as the means of endophytic transportation (James et al. 2002, Compant et al. 2005). The endodermis protects the vascular tissue from microbial invasion and needs to be breached by endophytes, which can take place through an active degradation of cell walls (Kovtunovych et al. 1999, Compant et al. 2005). *M. extorquens* DSM13060 was able to penetrate through both epidermis and endodermis, reaching the xylem within weeks (Fig. 8). Emergence of bacteria within the meristematic cells next to xylem vessels in the apex suggests that the endosymbiont reaches the niche of buds through the vascular system from the roots. However, the colonization of buds can have a different pattern in mature trees.

Colonization of pine by *M. extorquens* DSM13060 was relatively slow, measured in days instead of hours, compared to previously reported plant symbionts (d’Haeze et al. 2003, Compant et al. 2005) or pathogens (Martín-Rodrigues et al. 2013, Mensi et al. 2014). Although the bacterium heavily colonized the cylindrical sheath, epidermis, and cortex, the number of bacteria was low in the inner plant tissues. This was also observed in the seedlings of the final sampling at 730 dpi, where bacterial cells were sparsely spread in the non-vascular parenchyma of the roots (Supplementary Fig. S1a). Our earlier results have shown that the host plant can control the access by *Methylobacterium* endophytes to the plant interior (Ardanov et al. 2012).

### *MxaF*-promoter is most active on the plant surfaces

Plants produce methanol mainly as a by-product of de-esterification reactions of cell-wall pectin (Fall and Benson 1996, Micheli 2001). Formation of new cells and tissues produce high quantities of methanol, up to 27 μg g^−1^ fresh weight (Nemecek-Marshall et al. 1995, Hüve et al. 2007). Methanol can be toxic and is mainly emitted (Ramírez et al. 2006), being one of the main volatiles released from pine forests (Fehsenfeld et al. 1992). Plants are unable to fix methanol and support a rich methylotrophic community on the phyllosphere (Delmotte et al. 2009, Chistoserdova and Lidstrom 2013).

Previous studies have revealed that the *mxaF* promoter, induced by methanol, is highly active in *M. nodulans* during epiphytic and nodule-bound lifestyles (Jourand et al. 2005, Sy et al. 2005). Inactivation of bacterial methylotrophy reduces the competitive fitness of epiphytic populations (Sy et al. 2005) and has a negative effect on host-plant growth (Jourand et al. 2005). Although the promoter-reporter assays on *mxaF* provide only indirect evidence on methanol assimilation (Skovran et al. 2010), such experiments are superior in assessment of symbiotic bacterial metabolism *in planta*. We used the mCherry fluorescent reporter controlled by the *mxaF* promoter to gain novel information on methanol utilization during the colonization by the endosymbiont *M. extorquens* DSM13060. Similar to *M. extorquens* AM1, the endosymbiont possesses the Ln/Ce-dependent methanol dehydrogenase *XoxF (*Koskimäki et al. 2015), which could theoretically contribute to the observed methanol fixation. However, *M. extorquens* 13061 colonized seedlings grown in the chemically inert vermiculite that lacks lanthanides (e.g. La^3+^), therefore rendering *XoxF* transcriptionally inactive and catalytically impaired (Schmidt et al. 2010, Skovran et al. 2011).

Studies based on direct measurement of methanol emission had hypothesized methanol concentrations to be high inside the plant (MacDonald and Fall 1993). However, our results indicate that bacterial methanol consumption is especially high on plant surfaces and low in the plant interior. *M. extorquens* DSM13060 colonies showed the highest *mxaF*-induced mCherry fluorescence on the germinating root surface. Activity of the *mxaF* promoter suggests that methanol is efficiently used as an energy source in the outer tissues, epidermis, and cylindrical sheath (≤100 dpi).

A clear variation in the activity of *mxaF* expression was detected between individual bacterial cells, indicating a controlled orchestration of carbon utilization inside the bacterial colonies. Overlapping localization of the *mxaF* activity and the PHB granules suggests directing of the metabolic carbon flow to the biosynthesis of PHB, shown previously by our labeling studies (Koskimäki et al. 2016). The importance of PHB accumulation for bacteria during plant colonization has been reported in several studies (Aneja et al. 2004, Ratcliff et al. 2008). Similar to *M. extorquens* DSM13060, PHB is present in rhizobial cells invading the plant tissue through infection threads and becomes degraded during the progress of invasion (Hirsch et al. 1983). The role of PHB was earlier mainly considered as a storage molecule in the presence of excess carbon (Bourque et al. 1995). However, our previous results showed that upon infection of the host, the methanol-derived PHB becomes degraded by *M. extorquens* DSM13060 into methyl-esterified 3-hydroxybutyrate oligomers, which possess potent antioxidant activity. The activity protects bacteria from host-produced reactive oxygen species during colonization (Koskimäki et al. 2016, Müller-Santos et al. 2021). Therefore, our results suggest that methanol available on the host peripheral tissues serves as a raw material for compounds that enable penetration of further tissues.

Based on the activity of the *mxaF* promoter, methanol is abundant in the rhizosphere during the first months of seedling growth, but when the secondary growth begins, availability of methanol changes. Intracellular *M. extorquens* DSM13060 aggregates were common in the parenchyma surrounding the vascular tissues from 80 dpi onwards with no detectable *mxaF* activity. Based on the *mxaF* promoter analysis, methanol was scarce or completely unavailable in the inner cortex, non-vascular parenchyma, and chlorenchyma, where dividing bacterial cells were present. The active proliferation in the absence of methanol indicates that the endosymbiont utilizes another carbon source in these tissues, potentially malate (Koskimäki et al. 2022), similar to rhizobia (Mitsch et al. 2018).

## Conclusion

*M. extorquens* DSM13060 colonization is not limited to buds but is systemic in Scots pine seedlings, with cylindrical sheath and stomata on the stem serving as entry points. The colonization process has many unique features, such as penetration of plant without tissue breakage and propagation within living cells. Our results suggest that the endosymbiont fixes methanol mainly on plant surface to enter plant tissue. The similarities of colonization shared with rhizobia, such as infection pocket and thread formation, suggest that the endosymbiotic interaction of *M. extorquens* DSM13060 with pine is a mutual and intimate process.

## Materials and Methods

### Bacterial strains and culture conditions

The wild-type *M. extorquens* DSM13060 and its transgenic derivative *M. extorquens* 13061 generated by (Pohjanen et al. 2014), having stable genomic insert of two successive genes encoding GFP under constitutive promoter of *nptII* were used. For studying methanol utilization by *M. extorquens* DSM13060 during interaction with Scots pine, a reporter construct controlled by the promoter of methanol dehydrogenase α subunit (*mxaF*) (GenBank accession KM116512) was created. *M. extorquens* 13061 was used as a base strain to create the fluorescent mCherry biosensor strain, *M. extorquens* 13061-*mxaF*. Construction of the reporter strain as well as the bacterial strains, plasmids, and primers used are described in Supplementary Methods S1, Supplementary Tables S1 and S2. Bacteria were routinely grown at 28°C in M9 minimal salts media supplemented with 120 mM methanol and 18.5 mM of sodium succinate or in ammonium mineral salts (AMS) media supplemented with 30 mM of sodium succinate and appropriate antibiotics.

### Plant material, growth conditions, and inoculation

Scots pine seeds were collected from Pudasjärvi, Finland (65°05′N—65°28′N, 26°09′E—27°43′E). Seeds were treated at 55°C 72 h, incubated in sterile water at RT overnight, surface-sterilized with 3% calcium hypochlorite for 15 min, rinsed thoroughly with sterile water and planted in sterile glass jars containing moist vermiculite. The seeds were allowed to germinate for 5 days in growth chambers at 25 ± 1°C, 16/8 h photoperiod under fluorescent tubes (Philips TL-D 840) with light intensity of 40 μmol m^−2^ s^−1^. The seeds were inoculated with *M. extorquens* 13061 or 13061-*mxaF* by pipetting 100 μl of 2.5 × 10^7^ CFU/ml bacterial suspension on each germinating seed. The seedlings were grown for up to 5 months in 16/8 h photoperiod at 25 ± 1°C. After 5 months, selected seedlings were transferred to a 10-l sterile growth chamber filled with sterile pre-fertilized nursery peat (Finnpeat, Kekkilä Oyj, Finland) and grown under the same conditions for the final sampling.

### Confocal laser scanning microscopy

For the confocal laser scanning microscopy (CLSM) and acridine orange–ethidium bromide (AO-EB) staining (see Supplementary Methods S1), seedlings were collected twice a week for the first month and once a week for the second and third month. Samplings were continued twice a month until seedlings were 5 months old (150 dpi). One final sampling was performed at 730 dpi (24 months). At harvest, five individual seedlings (*n* = 5) were randomly selected and roots were rinsed with sterile water. Roots, shoots, needles, and buds were excised to 3-mm pieces and fixed as described by (Koskimäki et al. 2015). The samples were cut into 20–30 μm sections with a cryomicrotome (Reichert-Jung 2800 Frigocut with 2040 microtome) and mounted on slides with ProLong Gold Antifade Reagent (Invitrogen, Carlsbad, CA, USA). The sections were studied with CLSM (LSM 5 Pascal, Carl Zeiss, Germany) as described by (Koskimäki et al. 2015). Briefly, different excitation and emission wavelengths were utilized for GFP, mCherry, and endogenous autofluorescence of the plant tissues. For multichannel images of GFP and mCherry, HFT 488/543/633 nm beam splitter was used with secondary dichromic mirror NFT 545 to discriminate between the emissions. The projections of both channels were analyzed and merged using Zeiss LSM Image Browser (ver. 4.2.0.121, Carl Zeiss, Germany). Videos were processed and prepared from merged confocal z-stack images with Zen lite 2012 software (Carl Zeiss).

### Detection of PHB

*M. extorquens* DSM13060 cells were harvested by centrifugation (5000 × *g*, 5 min, 4°C), mixed with 4% (w/v) paraformaldehyde in PBS pH 7.4 and fixed at 4°C for 12 h. Cells were washed with PBS and stained with 1% (w/v) aqueous Nile blue A solution at 55°C for 10 min as described by Ostle and Holt (1982), washed with PBS, re-suspended in 0.1 M Na-phosphate buffer (pH 7.4) with 10% glycerol (v/v), and mounted on slides coated with 2% (w/v) agar. The same protocol was adapted for pine roots. Roots of mock-inoculated control and *M. extorquens* DSM13060-inoculated seedlings were cut to 3-mm segments 60 days post-inoculation (dpi) and fixed with 4% paraformaldehyde (w/v), 0.1% glutaraldehyde (v/v), 20% glycerol (v/v) and 0.1 M Na-phosphate buffer (pH 7.4) for 4 h under vacuum at 4°C. The samples were then stained and de-stained as described above, cut into 25–35 μm sections by cryomicrotome and mounted on slides with 0.1 M Na-phosphate buffer (pH 7.4), 10% glycerol (v/v). The bacterial cells and root sections were studied with LSM 5 Pascal. The Nile blue A-stained PHB granules were excited by an Argon Ion laser at 514 nm and emission was detected through a 560-to-615-nm BP filter. The background fluorescence of the bacterial cells was exited at 633 nm by HeNe laser and detected with a 650-nm LP filter. For the root samples, the HeNe laser was used for excitation of the Nile blue A-stained PHB granules at 514 nm, and the emission was detected through a 560-to-615-nm BP filter to minimize the autofluorescence from plant tissue. Endogenous autofluorescence of the plant cell walls, altered by Nile blue A staining, was used to illustrate the outline of the root morphology by excitation at 633 nm by HeNe laser and detection using a 650-nm LP filter. Due to excessive fluorescence from chloroplasts or Nile blue A-stained starch granules, the protocol was not applicable to detection of PHB in the aboveground tissues or older seedlings. For multi-channel images of Nile blue A-stained PHB granules and root autofluorescence, an HFT 488/543/633-nm beam splitter was used with a secondary NFT 635 dichromic mirror to discriminate between the emissions. The CLSM settings were kept equivalent for all samples for consistent results. The projections of multichannel images were processed and analyzed using ZEN lite 2012 (Blue edition; Carl Zeiss).

### Field emission scanning electron microscopy

Pine seedlings were inoculated with *M. extorquens* DSM13060 and samples were fixed with 4% paraformaldehyde, 0.1% glutaraldehyde in 0.1 M sodium buffer pH 7.4 for 9 h under vacuum at 4°C, rinsed with PBS and dehydrated through an alcohol series. For SEM studies, samples were subjected to critical-point drying with carbon dioxide in BAL-TEC CPD 030 (BAL-TEC Ltd., Balzers, Liechtenstein) and sputter-coated with platinum before imaging by field emission scanning electron microscopy (FE-SEM) (Carl Zeiss Sigma, Öberkochen, Germany) at an accelerating voltage of 5 kV.

### Determination of counts of living bacteria

The epi- and endophytic bacteria were determined at 15 dpi and 60 dpi from in vitro seedlings, inoculated with *M. extorquens* 13061 as described above. Seedlings were removed from the vermiculite, and roots were gently rinsed in sterile distilled water. The seedlings were surface-sterilized with 1.5% calcium hypochlorite for 10 min and rinsed thoroughly with sterile water. The seedlings were incised, and the roots, transition zones, stems, and needles of three seedlings were combined together. The preparation of samples and bacterial determination was conducted in sterile conditions following the protocol described by (Compant et al. 2005) with minor modifications. Each sample was ground in a mortar containing 1 ml of PBS, the homogenate was filtered, serially diluted 10-fold, and cultured on AMS plates supplemented with appropriate antibiotics. Bacterial colonies were counted after 5 days of incubation at 28°C.

## Supplementary Material

pcp-2025-e-00132-File014_pcaf135

pcp-2025-e-00132-File010

pcp-2025-e-00132-File011

pcp-2025-e-00132-File012

pcp-2025-e-00132-File013

## Data Availability

All data is available within the paper or supplementary materials. Supplementary data is available online. Reprints and permissions information are available online. Correspondence and requests for materials should be addressed to J.J.K.

## References

[ref1] Anand, R. and Chanway, C.P. (2013) Detection of GFP-labeled *Paenibacillus polymyxa* in autofluorescing pine seedling tissues. Biol. Fertil. Soils 49:111–118.

[ref2] Aneja, P., Dai, M., Lacorre, D.A., Pillon, B., Charles, T.C. (2004) Heterologous complementation of the exopolysaccharide synthesis and carbon utilization phenotypes of *Sinorhizobium meliloti* Rm1021 polyhydroxyalkanoate synthesis mutants. FEMS Microbiol. Lett. 239:277–283. 15476977 10.1016/j.femsle.2004.08.045

[ref3] Ardanov, P., Sessitsch, A., Häggman, H., Kozyrovska, N., Pirttilä, A.M. (2012) Methylobacterium-induced endophyte community changes correspond with protection of plants against pathogen attack. PLoS One 7:e46802. 23056459 10.1371/journal.pone.0046802 PMC3463518

[ref4] Bell, C.R., Dickie, G.A., Harvey, W.L.G., Chan, J.W.Y.F. (1995) Endophytic bacteria in grapevine. Can. J. Microbiol. 41:46–53.

[ref5] Bonaldi, K., Gargani, D., Prin, Y., Fardoux, J., Gully, D., Nouwen, N., et al. (2011) Nodulation of *Aeschynomene afraspera* and *A. indica* by photosynthetic *Bradyrhizobium* sp. strain ORS285: the nod-dependent versus the nod-independent symbiotic interaction. Mol. Plant-Microbe Interact. 24:1359–1371. 21995799 10.1094/MPMI-04-11-0093

[ref6] Bourque, D., Pomerleau, Y., Groleau, D. (1995) High-cell-density production of poly-β-hydroxybutyrate (PHB) from methanol by *Methylobacterium extorquens*: production of high-molecular-mass PHB. Appl. Microbiol. Biotechnol. 44:367–376.

[ref7] Capoen, W., Goormachtig, S., Holsters, M. (2010) Water-tolerant legume nodulation. J. Exp. Bot. 61:1251–1255. 19933316 10.1093/jxb/erp326

[ref8] Carrell, A.A. and Frank, A.C. (2014) *Pinus flexilis* and *Picea engelmannii* share a simple and consistent needle endophyte microbiota with a potential role in nitrogen fixation. Front. Microbiol. 5:333. 25071746 10.3389/fmicb.2014.00333 PMC4082182

[ref9] Chistoserdova, L. and Lidstrom, M.E. (2013) Aerobic methylotrophic prokaryotes. In: The Prokaryotes. Edited by Rosenberg, E., DeLong, E.F., Lory, S., Stackebrandt, E. and Thompson, F. pp 267–285. Springer Reference, 4th Ed., Springer-Verlag Berlin Heidelberg, Germany.

[ref10] Compant, S., Reiter, B., Sessitsch, A., Nowak, J., Clément, C., Ait, B.E. (2005) Endophytic colonization of *Vitis vinifera* L. by plant growth-promoting bacterium *Burkholderia* sp. strain PsJN. Appl. Environ. Microbiol. 71:1685–1693. 15811990 10.1128/AEM.71.4.1685-1693.2005 PMC1082517

[ref11] D’Haeze, W., Gao, M., De Rycke, R., Van Montagu, M., Engler, G., Holsters, M. (1998) Roles for azorhizobial nod factors and surface polysaccharides in intercellular invasion and nodule penetration, respectively. Mol. Plant-Microbe Interact. 11:999–1008.

[ref12] d’Haeze, W., De Rycke, R., Mathis, R., Goormachtig, S., Pagnotta, S., Verplancke, C., et al. (2003) Reactive oxygen species and ethylene play a positive role in lateral root base nodulation of a semiaquatic legume. Proc. Natl. Acad. Sci. 100:11789–11794. 12975522 10.1073/pnas.1333899100 PMC208836

[ref13] Delmotte, N., Knief, C., Chaffron, S., Innerebner, G., Roschitzki, B., Schlapbach, R., et al. (2009) Community proteogenomics reveals insights into the physiology of phyllosphere bacteria. Proc. Natl. Acad. Sci. 106:16428–16433. 19805315 10.1073/pnas.0905240106 PMC2738620

[ref14] Dong, Y., Iniguez, A.L., Ahmer, B.M.M., Triplett, E.W. (2003) Kinetics and strain specificity of rhizosphere and endophytic colonization by enteric bacteria on seedlings of *Medicago sativa* and *Medicago truncatula*. Appl. Environ. Microbiol. 69:1783–1790. 12620870 10.1128/AEM.69.3.1783-1790.2003 PMC150109

[ref15] Fall, R. and Benson, A.A. (1996) Leaf methanol—the simplest natural product from plants. Trends Plant Sci. 1:296–301.

[ref16] Fehsenfeld, F., Calvert, J., Fall, R., Goldan, P., Guenther, A.B., Hewitt, C.N., et al. (1992) Emissions of volatile organic compounds from vegetation and the implications for atmospheric chemistry. Glob. Biogeochem. Cycles 6:389–430.

[ref17] Germaine, K., Keogh, E., Garcia-Cabellos, G., Borremans, B., van der Lelie, D., Barac, T., et al. (2004) Colonisation of poplar trees by gfp expressing bacterial endophytes. FEMS Microbiol. Ecol. 48:109–118. 19712436 10.1016/j.femsec.2003.12.009

[ref18] Goormachtig, S., Capoen, W., Holsters, M. (2004) Rhizobium infection: lessons from the versatile nodulation behaviour of water-tolerant legumes. Trends Plant Sci. 9:518–522. 15501175 10.1016/j.tplants.2004.09.005

[ref19] Hardoim, P.R., van Overbeek, L.S., Berg, G., Pirttilä, A.M., Compant, S., Campisano, A., et al. (2015) The hidden world within plants: ecological and evolutionary considerations for defining functioning of microbial endophytes. Microbiol. Mol. Biol. Rev. 79:293–320. https://pubmed.ncbi.nlm.nih.gov/26136581. 26136581 10.1128/MMBR.00050-14 PMC4488371

[ref20] Harrison, M.J. (1999) Molecular and cellular aspects of the arbuscular mycorrhizal symbiosis. Annu. Rev. Plant Biol. 50:361–389. 10.1146/annurev.arplant.50.1.361 15012214

[ref21] Hirsch, A.M., Bang, M., Ausubel, F.M. (1983) Ultrastructural analysis of ineffective alfalfa nodules formed by nif::Tn5 mutants of *Rhizobium meliloti*. J. Bacteriol. 155:367–380. 6575011 10.1128/jb.155.1.367-380.1983 PMC217689

[ref22] Hüve, K., Christ, M.M., Kleist, E., Uerlings, R., Niinemets, Ü., Walter, A., et al. (2007) Simultaneous growth and emission measurements demonstrate an interactive control of methanol release by leaf expansion and stomata. J. Exp. Bot. 58:1783–1793. 17374874 10.1093/jxb/erm038

[ref23] Izumi, H. (2011) Diversity of endophytic bacteria in forest trees. In: Endophytes of Forest Trees: Biology and Applications. Edited by Pirttilä, A.M. and Frank, A.C. pp 95–105. Springer, Dordrecht.

[ref24] James, E.K., Reis, V.M., Olivares, F.L., Baldani, J.I., Döbereiner, J. (1994) Infection of sugar cane by the nitrogen-fixing bacterium *Acetobacter diazotrophicus*. J. Exp. Bot. 45:757–766.

[ref25] James, E.K., Olivares, F.L., Baldani, J.I., Döbereiner, J. (1997) Herbaspirillum, an endophytic diazotroph colonizing vascular tissue 3 Sorghum bicolor L Moench. J Exp Bot 48:785–798.

[ref26] James, E.K., Gyaneshwar, P., Mathan, N., Barraquio, W.L., Reddy, P.M., Iannetta, P.P.M., et al. (2002) Infection and colonization of rice seedlings by the plant growth-promoting bacterium *Herbaspirillum seropedicae* Z67. Mol. Plant-Microbe Interact. 15:894–906. 12236596 10.1094/MPMI.2002.15.9.894

[ref27] Jourand, P., Renier, A., Rapior, S., de Faria, S.M., Prin, Y., Galiana, A., et al. (2005) Role of methylotrophy during symbiosis between *Methylobacterium nodulans* and *Crotalaria podocarpa*. Mol. Plant-Microbe Interact. 18:1061–1068. 16255245 10.1094/MPMI-18-1061

[ref28] Koskimäki, J.J., Pirttilä, A.M., Ihantola, E.-L., Halonen, O., Frank, A.C. (2015) The intracellular scots pine shoot symbiont *Methylobacterium extorquens* DSM13060 aggregates around the host nucleus and encodes eukaryote-like proteins. MBio 6:e00039–e00015. 25805725 10.1128/mBio.00039-15 PMC4453540

[ref29] Koskimäki, J.J., Kajula, M., Hokkanen, J., Ihantola, E.-L., Kim, J.H., Hautajärvi, H., et al. (2016) Methyl-esterified 3-hydroxybutyrate oligomers protect bacteria from hydroxyl radicals. Nat. Chem. Biol. 12:332–338. 26974813 10.1038/nchembio.2043

[ref30] Koskimäki, J.J., Pohjanen, J., Kvist, J., Fester, T., Härtig, C., Podolich, O., et al. (2022) The meristem-associated endosymbiont *Methylorubrum extorquens* DSM13060 reprograms development and stress responses of pine seedlings. Tree Physiol. 42:391–410. 34328183 10.1093/treephys/tpab102 PMC8842435

[ref31] Kovtunovych, G., Lar, O., Kamalova, S., Kordyum, V., Kleiner, D., Kozyrovska, N. (1999) Correlation between pectate lyase activity and ability of diazotrophic *Klebsiella oxytoca* VN 13 to penetrate into plant tissues. Plant Soil 215:1–6.

[ref32] MacDonald, R.C. and Fall, R. (1993) Detection of substantial emissions of methanol from plants to the atmosphere. Atmos. Environ. Part A 27:1709–1713.

[ref33] Martín-Rodrigues, N., Espinel, S., Sanchez-Zabala, J., Ortíz, A., González-Murua, C., Duñabeitia, M.K. (2013) Spatial and temporal dynamics of the colonization of *Pinus radiata* by *Fusarium circinatum*, of conidiophora development in the pith and of traumatic resin duct formation. New Phytol. 198:1215–1227. 23496340 10.1111/nph.12222

[ref34] Matías, L. and Jump, A.S. (2012) Interactions between growth, demography and biotic interactions in determining species range limits in a warming world: the case of *Pinus sylvestris*. For. Ecol. Manag. 282:10–22.

[ref35] McCutcheon, J.P., Boyd, B.M., Dale, C. (2019) The life of an insect endosymbiont from the cradle to the grave. Curr. Biol. 29:R485–R495. 31163163 10.1016/j.cub.2019.03.032

[ref36] Mensi, I., Vernerey, M.-S., Gargani, D., Nicole, M., Rott, P. (2014) Breaking dogmas: the plant vascular pathogen *Xanthomonas albilineans* is able to invade non-vascular tissues despite its reduced genome. Open Biol. 4:130116. 24522883 10.1098/rsob.130116 PMC3938051

[ref37] Micheli, F. (2001) Pectin methylesterases: cell wall enzymes with important roles in plant physiology. Trends Plant Sci. 6:414–419. 11544130 10.1016/s1360-1385(01)02045-3

[ref38] Mitsch, M.J., diCenzo, G.C., Cowie, A., Finan, T.M. (2018) Succinate transport is not essential for symbiotic nitrogen fixation by *Sinorhizobium meliloti* or *Rhizobium leguminosarum*. Appl. Environ. Microbiol. 84:e01561–e01517. 28916561 10.1128/AEM.01561-17 PMC5734035

[ref39] Müller-Santos, M., Koskimäki, J.J., Alves, L.P.S., de Souza, E.M., Jendrossek, D., Pirttilä, A.M. (2021) The protective role of PHB and its degradation products against stress situations in bacteria. FEMS Microbiol. Rev. 45:fuaa058. 33118006 10.1093/femsre/fuaa058

[ref40] Nemecek-Marshall, M., MacDonald, R.C., Franzen, J.J., Wojciechowski, C.L., Fall, R. (1995) Methanol emission from leaves (enzymatic detection of gas-phase methanol and relation of methanol fluxes to stomatal conductance and leaf development). Plant Physiol. 108:1359–1368. 12228547 10.1104/pp.108.4.1359 PMC157513

[ref40a] Ostle, A.G. and Holt, J.G. (1982) Nile blue A as a fluorescent stain for poly-beta-hydroxybutyrate. Appl. Environ. Microbiol. 44:238–241. 10.1128/aem.44.1.238-241.1982 PMC241995 6181737

[ref41] Pirttilä, A.M. and Frank, A.C. (2018) Endophytes of Forest Trees: Biology and Applications. Springer, Cham.

[ref42] Pirttilä, A.M., Laukkanen, H., Pospiech, H., Myllylä, R., Hohtola, A. (2000) Detection of intracellular bacteria in the buds of scotch pine (*Pinus sylvestris* L.) by in situ hybridization. Appl. Environ. Microbiol. 66:3073–3077. 10877808 10.1128/aem.66.7.3073-3077.2000 PMC92113

[ref43] Pirttilä, A.M., Joensuu, P., Pospiech, H., Jalonen, J., Hohtola, A. (2004) Bud endophytes of scots pine produce adenine derivatives and other compounds that affect morphology and mitigate browning of callus cultures. Physiol. Plant. 121:305–312. 15153198 10.1111/j.0031-9317.2004.00330.x

[ref44] Pirttilä, A.M., Pospiech, H., Laukkanen, H., Myllylä, R., Hohtola, A. (2005) Seasonal variations in location and population structure of endophytes in buds of scots pine. Tree Physiol. 25:289–297. 15631977 10.1093/treephys/25.3.289

[ref45] Pohjanen, J., Koskimäki, J.J., Sutela, S., Ardanov, P., Suorsa, M., Niemi, K., et al. (2014) Interaction with ectomycorrhizal fungi and endophytic Methylobacterium affects nutrient uptake and growth of pine seedlings in vitro. Tree Physiol. 34:993–1005. 25149086 10.1093/treephys/tpu062

[ref46] Ramírez, I., Dorta, F., Espinoza, V., Jiménez, E., Mercado, A., Peña-Cortés, H. (2006) Effects of foliar and root applications of methanol on the growth of Arabidopsis, tobacco, and tomato plants. J. Plant Growth Regul. 25:30–44.

[ref47] Ratcliff, W.C., Kadam, S., V, and Denison R.F. (2008) Poly-3-hydroxybutyrate (PHB) supports survival and reproduction in starving rhizobia. FEMS Microbiol. Ecol. 65:391–399. 18631180 10.1111/j.1574-6941.2008.00544.x

[ref48] Rincón, A., Ruiz-Díez, B., García-Fraile, S., García, J.A.L., Fernández-Pascual, M., Pueyo, J.J., et al. (2005) Colonisation of *Pinus halepensis* roots by *Pseudomonas fluorescens* and interaction with the ectomycorrhizal fungus *Suillus granulatus*. FEMS Microbiol. Ecol. 51:303–311. 16329878 10.1016/j.femsec.2004.09.006

[ref49] Ryan, R.P., Germaine, K., Franks, A., Ryan, D.J., Dowling, D.N. (2008) Bacterial endophytes: recent developments and applications. FEMS Microbiol. Lett. 278:1–9. 18034833 10.1111/j.1574-6968.2007.00918.x

[ref50] Schmidt, S., Christen, P., Kiefer, P., Vorholt, J.A. (2010) Functional investigation of methanol dehydrogenase-like protein XoxF in *Methylobacterium extorquens* AM1. Microbiology 156:2575–2586. 20447995 10.1099/mic.0.038570-0

[ref51] Skovran, E., Crowther, G.J., Guo, X., Yang, S., Lidstrom, M.E. (2010) A systems biology approach uncovers cellular strategies used by *Methylobacterium extorquens* AM1 during the switch from multi-to single-carbon growth. PLoS One 5:e14091. 21124828 10.1371/journal.pone.0014091 PMC2991311

[ref52] Skovran, E., Palmer, A.D., Rountree, A.M., Good, N.M., Lidstrom, M.E. (2011) XoxF is required for expression of methanol dehydrogenase in *Methylobacterium extorquens* AM1. J. Bacteriol. 193:6032–6038. 21873495 10.1128/JB.05367-11 PMC3194914

[ref53] Sy, A., Timmers, A.C.J., Knief, C., Vorholt, J.A. (2005) Methylotrophic metabolism is advantageous for *Methylobacterium extorquens* during colonization of *Medicago truncatula* under competitive conditions. Appl. Environ. Microbiol. 71:7245–7252. 16269765 10.1128/AEM.71.11.7245-7252.2005 PMC1287603

[ref54] Taghavi, S., Garafola, C., Monchy, S., Newman, L., Hoffman, A., Weyens, N., et al. (2009) Genome survey and characterization of endophytic bacteria exhibiting a beneficial effect on growth and development of poplar trees. Appl. Environ. Microbiol. 75:748–757. 19060168 10.1128/AEM.02239-08 PMC2632133

[ref55] Tillman-Sutela, E., Kauppi, A., Karppinen, K., Tomback, D.F. (2008) Variant maturity in seed structures of *Pinus albicaulis* (Engelm.) and *Pinus sibirica* (Du Tour): key to a soil seed bank, unusual among conifers? Trees 22:225–236.

[ref56] Timonen, S. (1995) Avoiding autofluorescence problems: time-resolved fluorescence microscopy with plant and fungal cells in ectomycorrhiza. Mycorrhiza 5:455–458.

[ref57] Weyens, N., Boulet, J., Adriaensen, D., Timmermans, J.-P., Prinsen, E., Van Oevelen, S., et al. (2012) Contrasting colonization and plant growth promoting capacity between wild type and a gfp-derative of the endophyte *Pseudomonas putida* W619 in hybrid poplar. Plant Soil 356:217–230.

