# Supplementary material for: A shoot endosymbiont colonizes pine host by unique and rhizobia-like mechanisms boosted by surface-fixed methanol
Source: Plant Cell Physiol. 2025 Oct 24;67(1):39–54. doi: 10.1093/pcp/pcaf135 (PMC12814881; doi:10.1093/pcp/pcaf135)
Supplement: pcp-2025-e-00132-File014_pcaf135 [file pcp-2025-e-00132-file014_pcaf135.pdf]

Supplementary Materials

Article title:

A shoot endosymbiont colonizes host by unique and rhizobia-like mechanisms boosted by surface-fixed methanol

Authors:

Janne J. Koskimäki[1*], Johanna Pohjanen[1], Emmi-Leena Ihantola[1,2], Suvi Sutela[1,3], Anna Maria Pirttilä[1*]

Affiliations:

[1]Ecology and Genetics Research Unit, University of Oulu, PO Box 3000, FIN-90014 Oulu, Finland; [2] Institute of Clinical Microbiology, University of Eastern Finland, PO Box 1627, FIN-70211 Kuopio, Finland; [3] Forest Health and Biodiversity, Natural Resources Institute Finland (Luke), Latokartanonkaari 9, FIN-00790 Helsinki, Finland; *Author for correspondence

The following Supplementary Material is available for this article:

Fig. S1 Endophytic persistence of *M. extorquens* DSM13060 in the Scots pine at 24 months post-inoculation.

Fig. S2 Colonization of the transition zone of Scots pine by *M. extorquens* DSM13060 at 15-100 days post-inoculation.

Fig. S3 *In situ* hybridization of bud tissue from mature Scots pine trees showing infection pockets and infection thread-like structures.

Fig. S4 *M. extorquens* DSM13060 permeating of the host cell, forming an intracellular aggregate and colonizing the adjacent cells in the transition zone of Scots pine at ≥ 90 days post-inoculation.

Fig. S5 Morphological identification of tissue viability of Scots pine colonized by *M. extorquens* DSM13060 at 80-100 days post-inoculation.

Fig. S6 Methanol assimilation and aggregate formation of *M. extorquens* DSM13060 during the colonization of Scots pine at 40-100 days post-inoculation.

Table S1 Oligonucleotide primers used for cloning and confirming the reporter strain in this study.

Table S2 Genotypes of bacterial strains and description of plasmids used in this study.

Table S3 The viable epi- and endophytic cell content of *M. extorquens* DSM13060 in roots, transition zone, stem, and needles of Scots pine at 15 and 60 days post-inoculation.

Video S1 Merged confocal z-stack images showing tightly packed endophytic infection pockets of *M. extorquens* DSM13060 in the root epidermis of Scots pine at ≥ 30 dpi.

Video S2 Merged confocal z-stack images showing endophytic *M. extorquens* DSM13060 cells in root tip of Scots pine at ≥ 40 dpi.

Video S3 Merged confocal z-stack images showing endophytic infection thread-like structures of *M. extorquens* DSM13060 in the vascular tissues of Scots pine root at ≥ 80 dpi.

Video S4 Merged confocal z-stack images showing endophytic *M. extorquens* DSM13060 in the xylem vessels of Scots pine root at ≥ 40 dpi.

Methods S1 Construction of bacterial reporter strains, acridine orange–ethidium bromide cell viability staining, and *in situ* hybridization.

Figure S1. Endophytic persistence of *M. extorquens* DSM13060 in the host *Pinus sylvestris* L. CLSM images of longitudinal and cross sections of root tissues at 730 dpi. The bacterial cells carrying a fluorescent GFP are visualized in bright green. (**a**) A root section, rich with biofilm-like bacterial growth (arrowheads) in the cortex near endodermis, and individual bacterial cells in the non-vascular parenchyma. (**b**, **c**) Bacteria in the shoot cortex and xylem vessels (arrowheads) colonizing the tissues in the same manner as observed at 100-120 dpi (Fig. 4g-i). (**d**) Endophytic bacteria in a large infection pocket (arrowheads) next to photosynthetic cells surrounding the vascular tissues of stem. (**e**) Endophytic, intracellular bacteria (arrowheads) in the xylem vessels and in the photosynthetic cells of chlorenchyma. (**f**) A merged image (left) and a GFP image (right) showing the root tissue with bacterial colonies on the epidermisis, abundant individual cells in the cortex, and the majority of endophytic bacteria (arrowheads) residing in the parenchymatous cells surrounding the xylem vessels. Microscopic sections: longitudinal (a); cross (b-f). Ch, chlorenchyma; Co, cortex; Cs, cylindrical sheath; E, epidermis; En, endodermis; NVp, non-vascular parenchyma; Xy, xylem. Scale bars; (a) 50 μm; (b-f) 10 μm.

Figure S1.

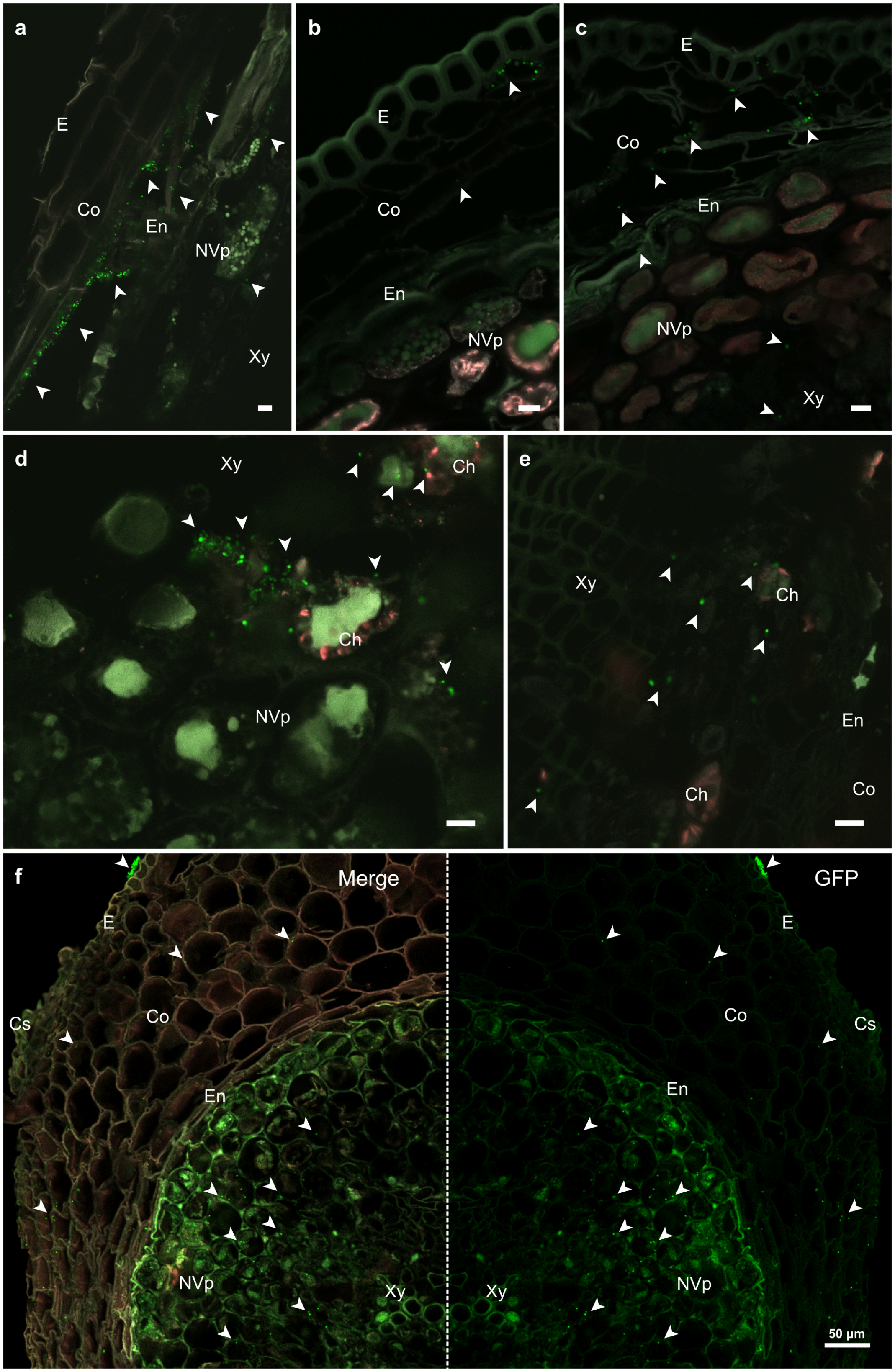
a
E
Co
En
NVp
Xy
b
E
Co
En
NVp
c
E
Co
En
NVp
Xy
d
Xy
Ch
Ch
NVp
e
Xy
Ch
En
Co
Ch
f
Merge
E
Cs
Co
En
NVp
Xy
GFP
E
Co
Cs
En
Xy
NVp
50 µm

Figure S2. Colonization of the transition zone of *Pinus sylvestris* L. by *M. extorquens* DSM13060. The bacterial cells carrying a fluorescent GFP are visualized in bright green. CLSM images of longitudinal and cross sections of (**a**) transition zone (Tz; black arrowheads) residing between root (R) and shoot (S) at 15-100 days post-inoculation (dpi). (**b**) An uninoculated control sample, showing the surface morphology of the outermost cell layer of cylindrical sheath, which covers large areas of the root, including the transition zone at 80 dpi. (**c**) In inoculated samples, the cylindrical sheath provided encasements for the bacterial colonization at 80 dpi. (**d**) The transition zone of the lower stem, where the outermost cells of the cylindrical sheath are invaded by bacteria at ≥ 15 dpi. (**e**) A below-ground tissue of transition zone rich with endophytic infection pockets observed in the cylindrical sheath and root epidermisis at 50 dpi. (**f**) Bacteria invaded the cell-layers of cylindrical sheath in masses by formation of biofilm-like structures, and progressed horizontally towards the cortex (arrow) at ≥ 60 dpi. Microscopic sections: longitudinal (b, c); cross (d-f). Co, cortex; Cs, cylindrical sheath; E, epidermisis; En, endodermis; NVp, non-vascular parenchyma Xy, xylem. Scale bars; 10 µm.

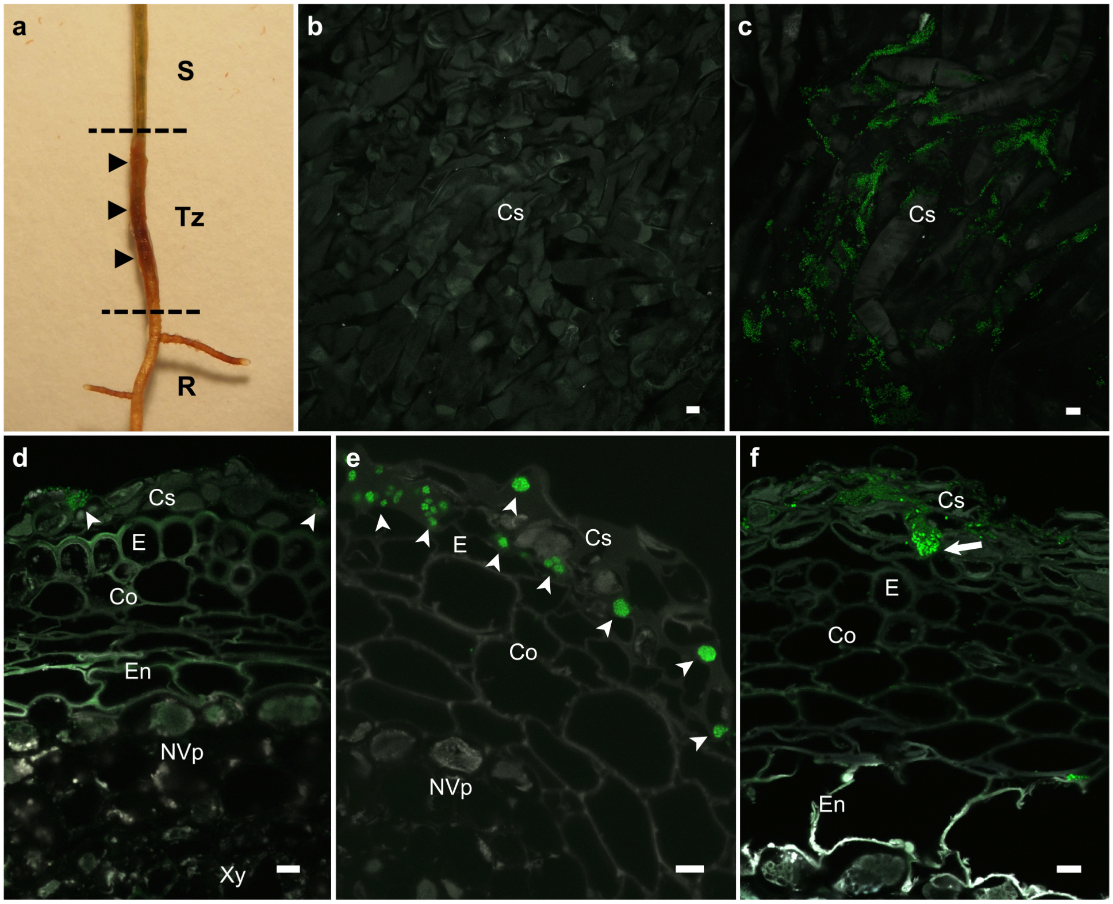

Figure S3. *In situ* hybridization of Scots pine bud tissue with digoxigenin-labeled oligonucleotide E11 (visualized in dark purple color). (**a**) An apical meristem (M) and scale primordia (S) of a bud where infection pockets (ip) and infection thread-like structures with a strong hybridization signal are marked (arrow). (**b**) Scale primordia (S) of another bud labeled with E11 showing numerous infection pockets (ip) and infection thread-like structures (arrows). ip, infection pocket; M, apical meristem; S, scale primordium. Scale bars, 20 μm.

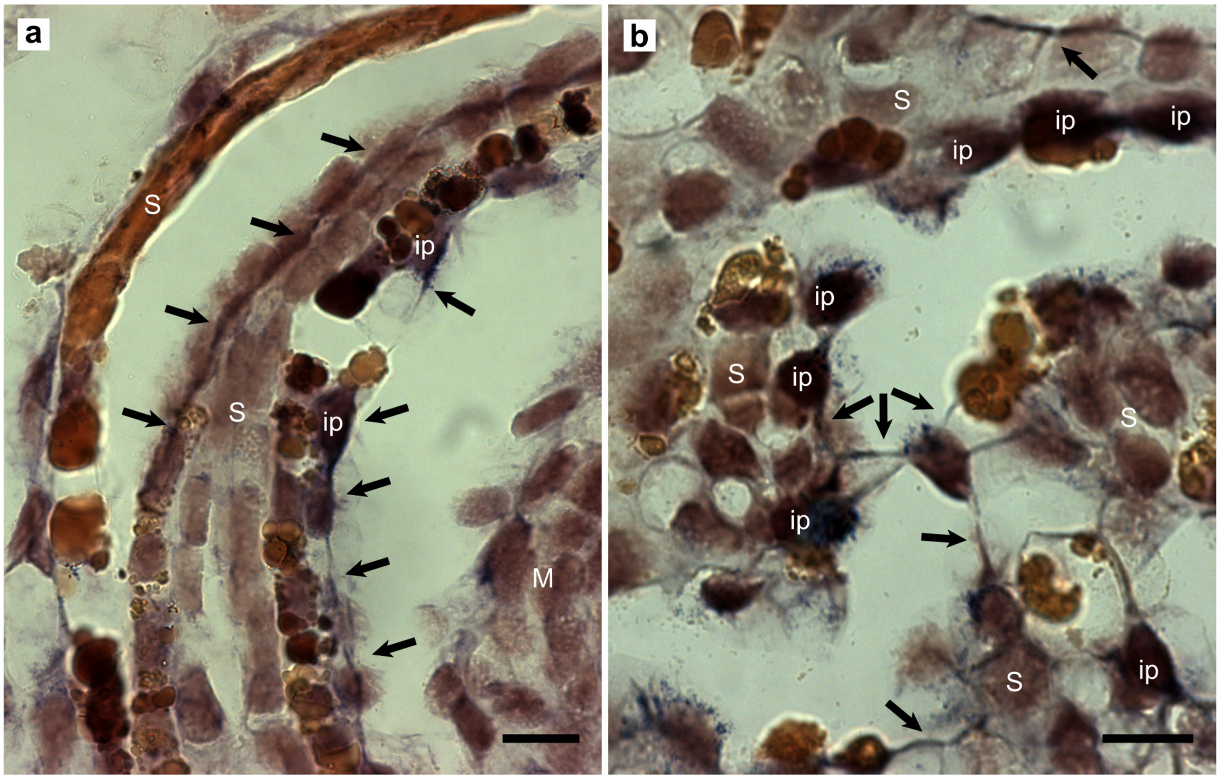

Figure S4. *M. extorquens* DSM13060 colonizing the tissues of the transition zone of *Pinus sylvestris* L. at ≥ 90 days post-inoculation (dpi). The bacterial cells carrying a fluorescent GFP are visualized in bright green. CLSM images of the consecutive longitudinal sections of z-stack. (**a, b**) The bacteria form an (1) intracellular aggregate at the (2) penetration site on the cell wall, which is followed by a permeation of the host cell and colonization of the adjacent cells. Co, cortex; E, epidermis; NVp, non-vascular parenchyma. Scale bars, 10 μm.

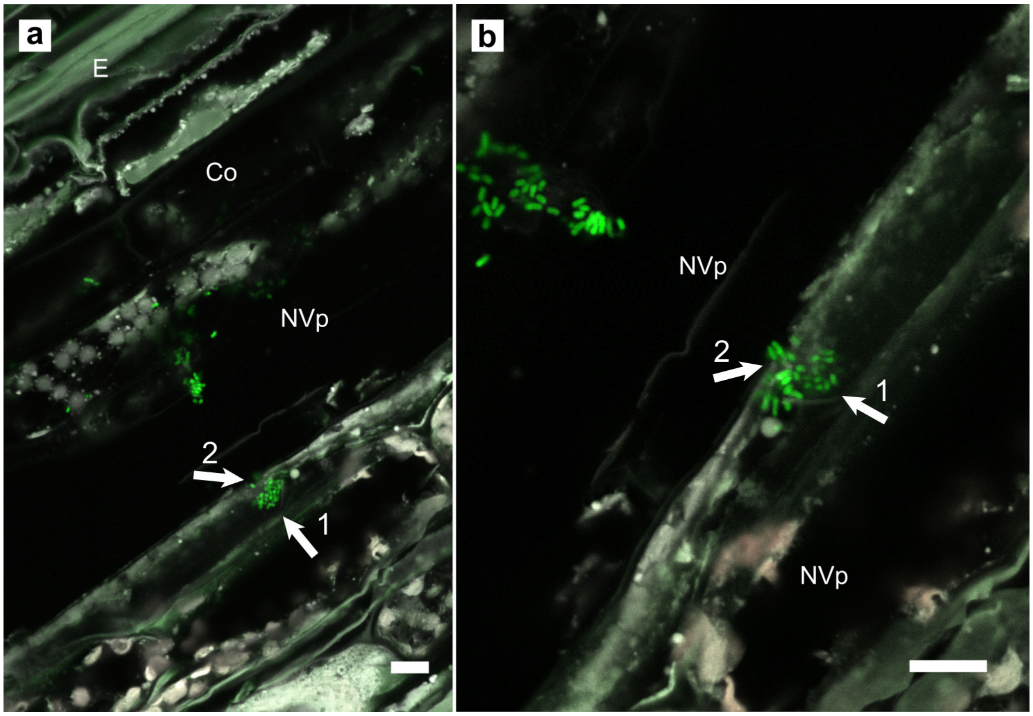

Figure S5. Morphological identification of tissue viability of *Pinus sylvestris* L. colonized by *M. extorquens* DSM13060. CLSM images of longitudinal and cross sections of root tissues were stained with acridine orange (AO) and ethidium bromide (EB) at 80-100 days post-inoculation (dpi). Note: The bacterial cells carrying a fluorescent GFP are visualized in green/white/purple due to the effects from AO-EB staining. (**a**) The spectrum of representative nuclei observed in the AO-EB stained tissue during the experiment. Morphology of nuclei with increased incorporation of EB (red) over AO (green) indicates the state of advancing programmed cell death (PCD) (black arrow). (**b**) An uninoculated, unstained control of the root tissue. (**c**) An inoculated, AO-EB stained negative control incubated in 20 mM H_2O_2, representing high background fluorescence and elevated levels of PCD. (**d**) An inoculated, AO-EB stained positive control incubated in PBS, showing characteristic nuclear morphology of a viable cell in the root tissues. (**e**) Bacteria colonizing healthy cells on the root surface, inside the root cortex (arrowheads), and inside healthy cells near nucleus (arrows). (**f**) Bacterial intra- and intercellular infection thread-like structures (arrowheads) in epidermis of root. (**g**) Large endophytic infection pockets (arrowheads) next to healthy cells of root cortex. (**h**) Endophytic bacteria (arrowheads) colonizing intact and living cells of the cylindrical sheath and a non-vascular parenchyma cell (arrow). (**i**) Bacteria (arrowheads) invading a viable cell to form infection pockets in endodermis of transition zone. (**j**) Individual bacterial cells (arrows) in the cytoplasms of healthy cortical cells. Microscopic sections: longitudinal (b-h, j); cross (i). Co, cortex; Cs, cylindrical sheath; E, epidermis; En, endodermis; n; nucleus; NVp, non-vascular parenchyma; Xy, xylem. Scale bars; 10 μm.

Figure S5.

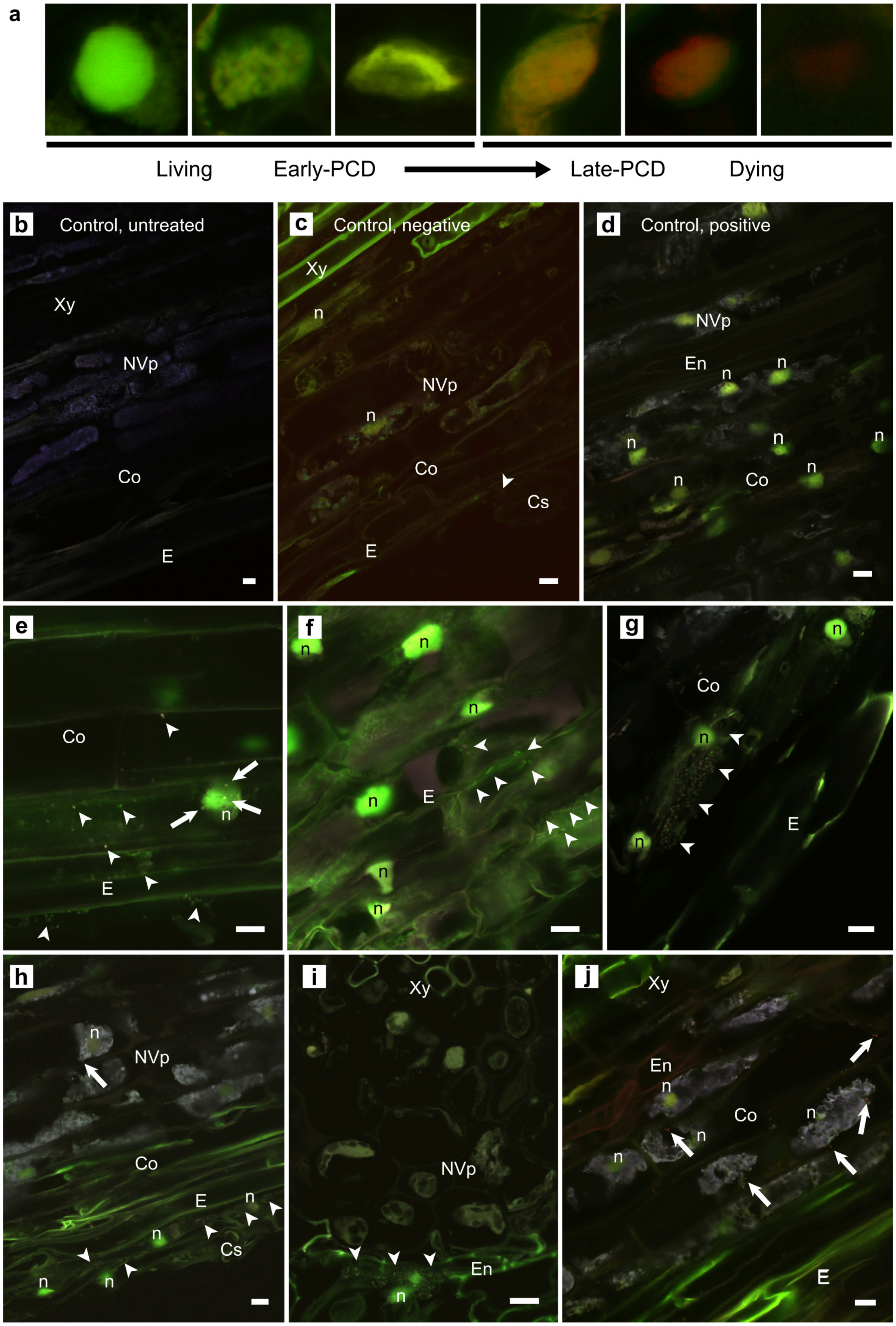
a
Living
Early-PCD
Late-PCD
Dying
b
Control, untreated
Xy
NVp
Co
E
c
Control, negative
Xy
n
NVp
n
Co
Cs
E
d
Control, positive
NVp
En
n
n
n
n
n
n
Co
n
e
Co
n
E
f
n
n
n
n
E
n
n
g
n
Co
n
E
n
h
n
NVp
Co
E
n
n
Cs
n
n
i
Xy
NVp
En
n
j
Xy
En
n
Co
n
n
n
E

Figure S6. Methanol assimilation and aggregate formation of *M. extorquens* DSM13060 during the colonization of *Pinus sylvestris* L. mCherry reporter controlled by the methanol inducible *mxaF* promoter (P*mxaF*::mCherry) was used as a biological sensor for bacterial methanol utilization. CLSM images of longitudinal and cross sections of root and shoot tissues at 40-100 days post-inoculation (dpi). Each panel includes a merged-image of a pine tissue section infected by bacteria with both tags, and by bacteria carrying the mCherry (white) or GFP tag (bright green). (**a**) Bacterial infection pockets (arrows) in the cylindrical sheath, displaying high-level methanol utilization at 40-60 dpi. (**b**) Endophytic infection pockets (arrows) in the cylindrical sheath and root epidermis at 50-70 dpi showing intermediate-level methanol assimilation. (**c**) Multicellular bacterial aggregate (arrow) in the stoma at 80-100 dpi displaying high-level methanol assimilation. (**d**) A tightly packed bacterial aggregate in a cortical cell without *mxaF* expression at 80-100 dpi. Microscopic sections: longitudinal (a, c, d); cross (b). Ch, chlorenchyma; Co, cortex; Cs, cylindrical sheath; E, epidermis; Gc, guard cell; NVp, non-vascular parenchyma; Xy, xylem. Scale bars; 10 μm.

Figure S6.

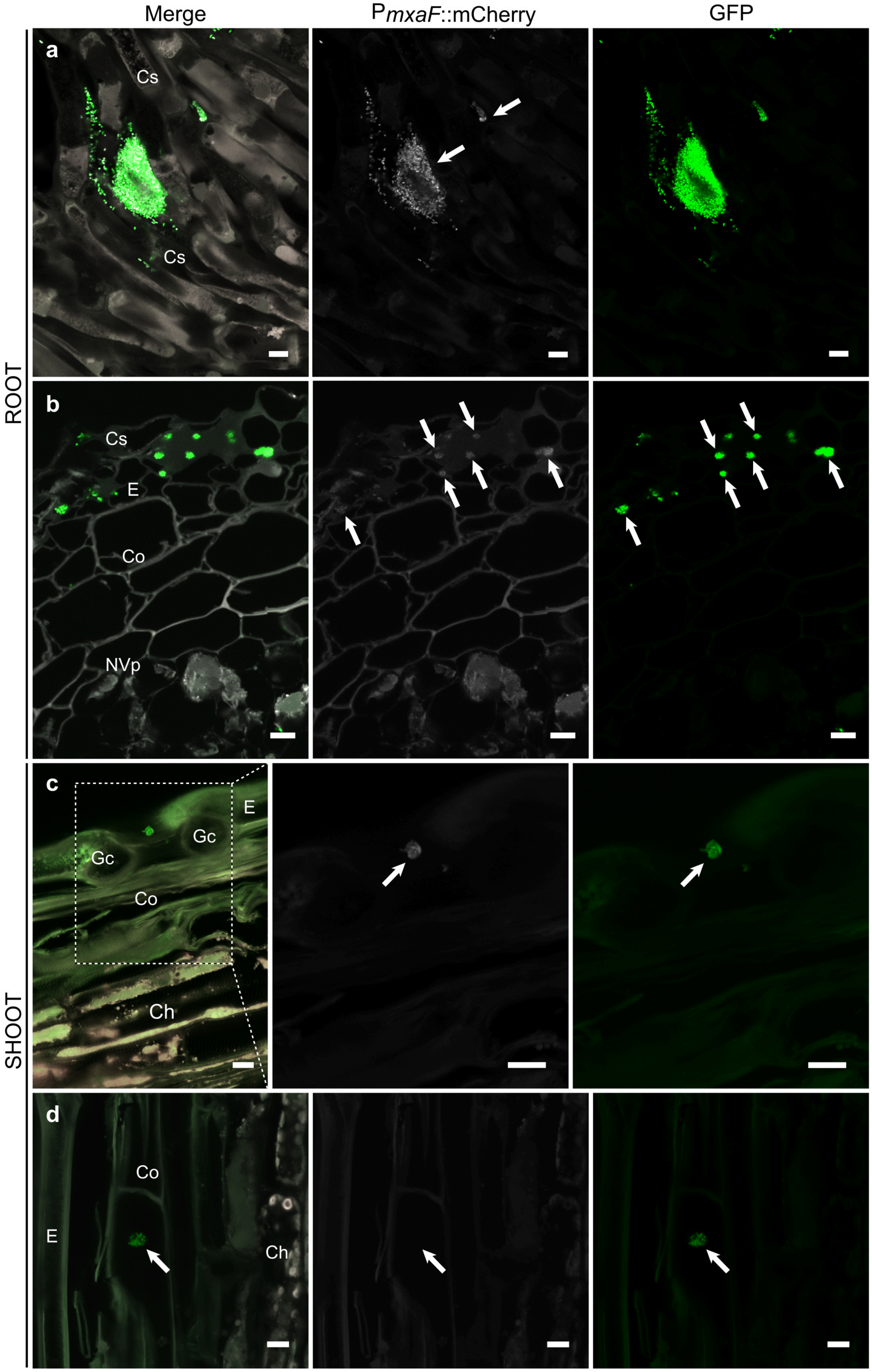
Merge
PmxaF::mCherry
GFP
ROOT
SHOOT
a
Cs
Cs
b
Cs
E
Co
NVp
c
E
Gc
Gc
Co
Ch
d
Co
E
Ch

Table S1. Oligonucleotide primers used for cloning and confirming the reporter strain in this study. F stands for forward primer and R for reverse primer. Restriction sites are underlined and overlapping sequences for OE-PCR are marked in italics.

Gene or plasmid	Sequence	Name, description
**For construction of *mxaF* promoter region fused with *mCherry***		
mxaF	TATTGGTACCATGGCCGACAAGCCTCCCGCTT	MxaFprom-F (*Kpn*I)
mxaF	*TCCTCGCCCTTGCTCACCAT*CCTGCGTCTCCTCGCTGAACCGCCT	MxaFprom-OE-R
mCherry	*GTTCAGCGAGGAGACGCAGG*ATGGTGAGCAAGGGCGAGGAG	OE-MxaF-mCherry-F
mCherry	TATTGGATCCTTTACTTGTACAGCTCGTCCATGC	mCherry-R2 (*Bam*HI)
**For sequencing constructs in plasmid pME6031**		
pME6031	CGTGATCGAAATCCAGATCC	pME6031-seq-F
	CGACTGAATCCGGTGAGAAT	pME6031-seq-R

Table S2. Genotypes of bacterial strains and description of plasmids used in this study.

Strain or plasmid	Description	Reference
Escherichia coli		
DH5α	*endA1 gyrSA96 hrdR17* (*rK-mK-*) *supE44 recA1*; general host strain used for transformation and propagation of plasmids	(Boyer & Roulland-Dussoix, 1969)
Methylobacterium extorquens		
DSM13060	Wild type; a plant growth-promoting conifer endophyte	(Pirttilä et al, 2000)
13061	DSM13060 containing mTn5*gusA-pgfp*21-*Km*^{r} cassette (pFAJ1820), *Km*^{r}	(Pohjanen et al, 2014)
13061-*mxaF*	Strain 13061 containing pMExt801 plasmid, *Tc*^{r}	This study
**Plasmids**		
pJET 1.2	Cloning vector for amplified PCR products; Ap^r	Thermo Fisher
pFAJ1820	pUT mini-Tn5 transposon carrying two copies of a GFP gene controlled by the *nptII* promoter, Km^r	(Xi et al. 1999)
pME6031	Broad host-range cloning vector which is maintained in Gram-negative bacteria without selection pressure, Tc^r	(Heeb et al, 2000)
pMP7604	pMP6031 derivate harboring mCherry gene under the control of the tac promoter, Tc^r	(Lagendijk et al, 2010)
pMExt671	pMP7604 pME6031 derivate harboring *mCherry* gene under control of *M.extorquens* DSM13060 *mxaF* promoter	This study

Table S3. The colony forming units (CFU) of *M. extorquens* 13061 in roots, transition zone, stem, and needles of *Pinus sylvestris* L. at 15 and 60 days post-inoculation (dpi). ND, bacteria not detected.

		M. extorquens **CFU/g**	
**Scots pine organ**	**dpi**	**Epi- and endophytic**	**Endophytic**
Root	15	2.16×10^{8}	ND
Transition zone	15	9.04×10^{7}	ND
Stem	15	7.25×10^{5}	ND
Needles	15	5.43×10^{6}	ND
Root	60	2.73×10^{8}	4.21×10^{4}
Transition zone	60	1.49×10^{8}	7.11×10^{6}
Stem	60	1.03×10^{7}	2.42×10^{4}
Needles	60	4.90×10^{5}	ND

Video S1. Merged confocal z-stack images showing tightly packed endophytic infection pockets of *M. extorquens* DSM13060 in the root epidermis of Scots pine at ≥ 30 dpi. The GFP-tagged bacterial cells are displayed in bright green, and the plant tissue morphology is visible in the green and red channels due to autofluorescence. The sections were collected at the focal interval of 1 µm (z-axis) depicted by the numbering at the top left corner. Images were processed and prepared with the Zen lite 2012 software (Blue edition, Carl Zeiss, Germany).

Video S2. Merged confocal z-stack images showing endophytic *M. extorquens* DSM13060 cells in root tip of Scots pine at ≥ 40 dpi. The white dashed lines highlight the GFP-tagged bacterial cells (bright green), and the plant tissue morphology is visible in the green and red channels due to autofluorescence. The sections were collected at the focal interval of 1 µm (z-axis) depicted by the numbering at the top left corner. Rc, root cap; Rm, root meristem. Images were processed and prepared with the Zen lite 2012 software (Carl Zeiss).

Video S3. Merged confocal z-stack images showing endophytic infection thread-like structures of *M. extorquens* DSM13060 during invasion of Scots pine vascular tissues in

the root at ≥ 80 dpi. The arrowheads highlight the infection thread-like structures of GFP-tagged bacterial cells (bright green), and the arrow shows the penetration site to the vascular tissues. The plant tissue morphology is visible in the green and red channels due to autofluorescence. The sections were collected at the focal interval of 1 µm (z-axis) depicted by the numbering at the top left corner. E, Epidermis; Co, cortex; En, endodermis; Xy, xylem. Images were processed and prepared with the Zen lite 2012 software (Carl Zeiss).

Video S4. Merged confocal z-stack images showing endophytic *M. extorquens* DSM13060 in the xylem vessels of Scots pine root at ≥ 40 dpi. The GFP-tagged bacterial cells are displayed in bright green, and the plant tissue morphology is visible in the green and red channels due to autofluorescence. The sections were collected at the focal interval of 1 µm (z-axis) depicted by the numbering at the top left corner. Xy, xylem; NVp, non-vascular parenchyma. Images were processed and prepared with the Zen lite 2012 software (Carl Zeiss).

Methods S1 Construction of bacterial reporter strains, cell viability staining, and *in situ* hybridization.

Construction of bacterial promoter-reporter strain

For the reporter construct, *mxaF* promoter region (370 bp) was amplified from genomic DNA of *M. extorquens* DSM13060 using Phusion High-Fidelity DNA polymerase (Thermo Fisher Scientific, Vantaa, Finland) with mxaFprom-F and OE-mxaFprom-R primers. A gene encoding the fluorescent reporter mCherry was amplified from the plasmid pMP7604 with primers OE-mxaF-mCherry-F and mCherry-R2 (Shaner *et al*., 2004; Lagendijk *et al*., 2010). The fragments were fused in an overlap extension PCR (OE-PCR) by mxaFprom-F and mCherry-R2 primers that had restriction sites for *Kpn*I and *BamH*I enzymes in the overhangs. The insert was propagated in *E.coli* DH5α and digested with *Kpn*I and *BamH*I restriction enzymes and constructs were ligated into the respective sites of pME6031 plasmid, to create plasmid pMExt671 (FastDigest; Thermo Scientific). Plasmid pME6031 is a broad-range low-copy vector (5 to 7 copies per cell)

that has been previously shown to be maintainable in plant-microbe interactions without selection pressure from antibiotics (Heeb *et al.*, 2000; Lagendijk *et al.*, 2010). The construct was sequenced using pME6031-seq-F and pME6031-seq-R primers with BigDye Terminator v.3.1 Cycle Sequencing Kit (Applied Biosystems, Foster City, CA, USA) and ABI 3730 automated sequencer (PE Applied Biosystem, USA). Sequences were analyzed with Sequencher® 4.7 (Gene Codes Corporation, Ann Arbor, MI, USA). Primer sequences with overhangs are described in Table S1. Bacterial strains and plasmids used in this study are described in Table S2. Reporter construct pMExt671 was electroporated into competent cells of *M. extorquens* 13061 using 0.1-cm gap cuvette and Gene Pulser I (BioRad, USA) apparatus with the following parameters: 25 μF, 200 Ω, 2 kV to the final field strength of 20 kV/cm. Selection of transformants was done on AMS medium containing 30 mM sodium succinate, tetracycline (16 μg/ml) and kanamycin (50 μg/ml). Stability of the transformants was determined with and without antibiotic selection pressure for over 20 generations. Methanol inducibility of the transformants with pMExt671 plasmid was verified by comparing the bacterial mCherry fluorescence under confocal microscope while incubated in sterile water and in M9 minimal medium supplemented with 120 mM MeOH. Furthermore, the colony and cell morphologies and growth patterns of the transformants were compared to the wild-type strain, as before (Pohjanen *et al.*, 2014), in Luria-Bertani, M9 minimal medium and preliminarily *in vitro* plant environment. The transformant selected for the colonization experiment was assigned *M. extorquens* pMExt671.

Identification of cell viability by acridine orange–ethidium bromide (AO-EB) staining

Double staining of the tissues with acridine orange (AO) and ethidium bromide (EB) allows distinguishing between live and dead cells, and enables differentiation among the stages of progressing programmed cell-death (PCD)(Renvoize *et al.*, 1998; Byczkowska *et al.*, 2013). AO emits green fluorescence when it binds to double-stranded nuclear chromatin. AO stains both live and dead cells, while EB can only penetrate cells that have lost cytoplasmic membrane integrity. In these cells, incorporation of EB can be detected as red fluorescence. Cells in the early-PCD have irregular nuclei stained to

green-yellow, where slightly condensed or fragmented chromatin is visible in the form of bright green patches. Cells with disrupted cytoplasmic membranes integrate EB to the chromatin in the late-PCD, which dominates over AO and gives cells an orange-red stain. Similar to healthy viable cells, necrotic cells have structurally normal morphology, but the nucleus is stained bright orange. Roots and lower stems (transition zones) of *in vitro*-cultured Scots pine seedlings inoculated with *M. extorquens* 13061 were sectioned as described in the main text (Confocal laser-scanning microscopy (CLSM)). Tissue sections were washed twice with phosphate-buffered saline (PBS) pH 7.4 and treated with AO-EB in PBS buffer (25 µg/ml each) in the dark for 15 min at room temperature (RT). Uninoculated root sections of pine seedlings were used as controls. The controls were incubated in 20 mM hydrogen peroxide (H_2O_2) for 2 hours (negative) and in PBS (positive) for the same duration of time prior to the AO-EB staining. After staining, the samples were rinsed three times with PBS. Fixation, cryosectioning and protocols involved with CLSM were performed as described in the main text (Confocal laser-scanning microscopy (CLSM)), using appropriate excitation and emission wavelengths (488 nm/BP 505-530 nm for AO; 514 nm/BP 560-615 nm for EB; 488 nm/LP 650 nm for plant cell autofluorescence). The settings were kept equivalent for all samples to obtain consistent results.

***In situ* hybridization**

Buds collected from mature Scots pine trees were surface sterilized: 1 min in 70% ethanol, 20 min in 6% calcium hypochlorite. After rinsing in sterile water, bud scales were removed aseptically. Buds were dissected longitudinally and fixed in 0.1M NaH_2PO_4–Na_2HPO_4 (pH 7.4), 2% paraformaldehyde and 2.5% glutaraldehyde at 4°C overnight. Tissues were dehydrated, cleared through an ethanol– t-butanol series, and embedded in paraffin (Merck, Whitehouse Station, NJ, USA). *In situ* hybridization was performed as described by Pirttilä *et al.* (2000) Briefly, the paraffin-embedded tissues were cut to 6-µm thick sections, baked on silane-coated slides, and paraffin was removed in xylene. Slides were air-dried, fixed, and treated with sodium borohydride and proteinase K, as described previously (Pirttilä *et al.*, 2000). The eubacterial probe E11, *Methylobacterium*-specific probe MB (Pirttilä *et al.*, 2000), and a control without probe were hybridized. The probes were labeled with digoxigenin using the DIG

Oligonucleotide 3′-End Labeling Kit (Roche). Hybridization was performed aseptically one to three times per bud sample. The slides were hybridized in a hybridization buffer containing 3× SET (450 mM NaCl, 60 mM Tris–HCl pH 7.5 and 3 mM EDTA), Denhardt's solution (0.02% Ficoll, Sigma-Aldrich, MO, USA, 0.02% polyvinyl pyrrolidone, Sigma-Aldrich, 0.02% bovine serum albumin, Sigma-Aldrich), 0.02% tRNA (Sigma-Aldrich), 0.02% polyadenylic acid (Sigma-Aldrich), 10% dextran sulfate (Merck), 50 mM dithiothreitol (Merck), formamide at the probe-specific concentration (Pirttilä *et al.*, 2000), and the probe (0.5 ng/µl). Slides containing the hybridization mixture were placed in a hybridization chamber saturated with 3× SET and incubated at 38°C overnight. After the hybridization, slides were washed with 2× SET at RT for 15 min and with 0.1× SET at 53°C for 15 min. Detection was performed with the DIG Nucleic Acid Detection kit (Roche Applied Science, Penzberg, Germany), after which the slides were rinsed with 70% ethanol, air-dried and viewed under bright-field microscope.

References

Boyer HW, Roulland-Dussoix D. 1969. A complementation analysis of the restriction and modification of DNA in *Escherichia coli*. *J Mol Biol* 41:459-472.

Byczkowska A, Kunikowska A, Kaźmierczak A. 2013. Determination of ACC-induced cell-programmed death in roots of *Vicia faba* ssp. minor seedlings by acridine orange and ethidium bromide staining. *Protoplasma* 250:121-128.

Heeb S, Itoh Y, Nishijyo T, Schnider U, Keel C, Wade J, *et al.* 2000. Small, stable shuttle vectors based on the minimal pVS1 replicon for use in gram-negative, plant-associated bacteria. *Mol Plant-Microbe Interact* 13:232-237.

Lagendijk EL, Validov S, Lamers GE, De Weert S, Bloemberg GV. 2010. Genetic tools for tagging Gram-negative bacteria with mCherry for visualization in vitro and in natural habitats, biofilm and pathogenicity studies. *FEMS Microbiol Lett* 305:81-90.

Pirttilä AM, Laukkanen H, Pospiech H, Myllylä R, Hohtola A. 2000. Detection of intracellular bacteria in the buds of Scotch pine (*Pinus sylvestris* L.) by in situ hybridization. *Appl Environ Microbiol* 66:3073-3077.

Pohjanen J, Koskimäki JJ, Sutela S, Ardanov P, Suorsa M, Niemi K, *et al.* 2014. Interaction with ectomycorrhizal fungi and endophytic *Methylobacterium* affects nutrient uptake and growth of pine seedlings in vitro. *Tree Physiol* 34:993-1005.

Renvoize C, Biola A, Pallardy M, Breard J. 1998. Apoptosis: identification of dying cells. *Cell Biol Toxicol* 14:111-120.
